# Restoration of the immune system with base editing and non-genotoxic conditioning in a *Rag2* point-mutant mouse model

**DOI:** 10.1016/j.ymthe.2026.04.010

**Published:** 2026-04-12

**Authors:** Carla Dib, Jack A. Queenan, Hana Willner, Leah Swartzrock, Carsten T. Charlesworth, Morgane Denis, Jessie R. Davis, Rina J. Mepani, Katie Ho, Madalena Castro, Ross C. Wilson, Hiromitsu Nakauchi, David R. Liu, Agnieszka Czechowicz

**Affiliations:** 1 Department of Pediatrics, Division of Hematology, Oncology, Stem Cell Transplantation and Regenerative Medicine, Stanford University School of Medicine, Stanford, CA 94304, USA; 2 Institute for Stem Cell Biology and Regenerative Medicine, Stanford University, Stanford, CA 94305, USA; 3 Merkin Institute of Transformative Technologies in Healthcare, Broad Institute of MIT and Harvard, Cambridge, MA 02142, USA; 4 Department of Chemistry and Chemical Biology, Harvard University, Cambridge, MA 02138, USA; 5 Howard Hughes Medical Institute, Harvard University, Cambridge, MA 02139, USA; 6 Innovative Genomics Institute, University of California, Berkeley, Berkeley, CA 94720, USA; 7 Department of Molecular and Cell Biology, University of California, Berkeley, Berkeley, CA 94720, USA; 8 California Institute for Quantitative Biosciences at University of California, Berkeley, Berkeley, CA 94720, USA

**Keywords:** gene therapy, base editing, engineered virus-like particles, hematopoietic stem cells, non-genotoxic conditioning, immunodeficiency

## Abstract

Transplantation of donor hematopoietic stem and progenitor cells (HSPCs) is a well-established curative treatment for various blood and immune diseases, including severe combined immunodeficiency (SCID). However, it comes with significant toxicities, including graft-versus-host disease (GvHD) and tissue damage resulting from the use of genotoxic chemotherapy-containing conditioning regimens. Autologous transplantation using gene-modified HSPCs eliminates GvHD but currently still relies on genotoxic conditioning. Further, gene modification of HSPCs has commonly utilized integrating viruses, which carry the risk of oncogenesis. The ideal therapy would eliminate the risks associated with current hematopoietic stem cell (HSC) gene-modification and conditioning approaches. Here, we combined base editors (BEs), engineered virus-like particles (eVLPs), and non-genotoxic αCD117 antibody-drug conjugate (ADC) conditioning to explore optimal curative treatment of SCID. We generated a *Rag2* SCID mouse model with a single point mutation (pm) and corresponding BE. *Rag2*^*pm/pm*^ HSPCs were corrected using SpCas9NG-ABE-eVLPs without off-target effects being detected. Even in settings of low editing, transplantation of BE-corrected HSPCs into αCD117-ADC-conditioned mice led to efficient immune cell production in peripheral blood with normal B cell progenitors in the bone marrow. Combining αCD117-ADC conditioning with transplantation of HSPCs that were base edited using eVLPs successfully reversed the SCID phenotype in mice, showcasing a significant advancement in reducing treatment-related toxicities while enabling disease correction.

## Graphical abstract

**Figure:**
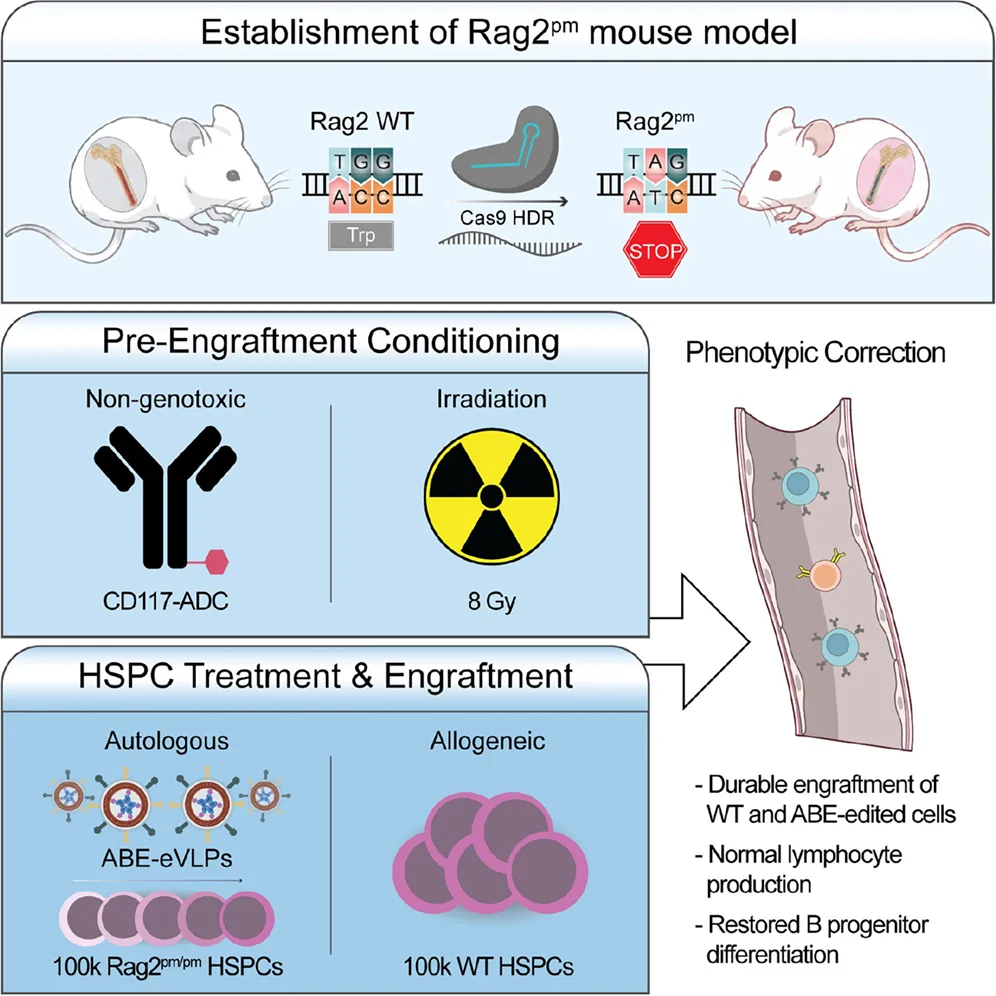

## Teaser

Dib and colleagues establish a novel point-mutant mouse model of severe combined immunodeficiency (SCID) and provide a framework for non-genotoxic disease correction using antibody-based conditioning and transplantation of autologous hematopoietic stem and progenitor cells (HSPCs) that have been gene modified using base editors delivered via engineered virus-like particles (eVLPs).

## Introduction

Primary immunodeficiency disorders (PIDs), also referred to as inborn errors of immunity (IEIs), encompass a wide range of conditions characterized by defects in the immune system. These diseases often result in recurrent or severe infections and autoimmune complications. The most serious PID is severe combined immunodeficiency (SCID), which is life threatening due to a combined absence of T cell and B cell function.^1^ While various treatments are available or in development for certain types of immunodeficiency,^2^ they often have limited efficacy^3^ and/or address only specific subtypes of PIDs.^4^ Currently, bone marrow or hematopoietic stem cell transplantation (BMT/HSCT) using hematopoietic stem and progenitor cells (HSPCs) from a healthy donor is the sole curative treatment option for many PIDs.^5^^,^^6^^,^^7^ However, despite its life-saving potential, allogeneic (allo) HSCT is associated with significant toxicities that can outweigh its benefits and therefore is utilized only in the treatment of acute PIDs such as SCID. These toxicities include immune-mediated tissue attack by the donor graft (graft-versus-host disease, GvHD) and severe tissue damage caused by current conditioning regimens, including genotoxic chemotherapy or irradiation (IR).^3^^,^^8^^,^^9^^,^^10^ Moreover, the lack of optimal HLA-matched familial donors further complicates the transplantation process for most patients.^8^^,^^10^ These challenges pose significant barriers to the widespread use of allo-HSCT, particularly for patients with immunodeficiencies that typically present in infancy.

The ideal approach to curing patients with PIDs would involve eliminating both major toxicities associated with current treatments. Autologous HSCT, which uses a patient’s own gene-modified HSPCs, offers two key benefits: it removes the need to search for a compatible donor and eliminates the risk of GvHD. Autologous HSCT using gene-modified HSPCs is being developed for many different blood and immune diseases and was initially pioneered in SCID.^11^ However, most approaches to date have relied on *ex vivo* gene modification of HSPCs using viral vectors, which carries a risk of oncogenesis.^6^^,^^12^^,^^13^^,^^14^ While advances in viral vectors from gamma-retroviruses to self-inactivating (SIN) lentiviral vectors have reduced the risk of random insertional mutagenesis,^15^^,^^16^ this risk remains, as evidenced by recent gene therapy trials.^17^

As an alternative to random gene insertion, recent precision gene engineering technologies have demonstrated the ability to directly correct genetic defects in HSPCs, including those causing SCID.^18^^,^^19^^,^^20^^,^^21^^,^^22^ However, a majority of the methods used in these studies integrate a DNA template or cause gene disruptions by inducing DNA double-strand breaks, which have been shown to result in upregulation of DNA damage response pathways,^23^^,^^24^ which may increase the likelihood of graft failure and/or oncogenesis. The CRISPR-Cas9 base-editing system offers a solution by precisely installing point mutations (pms) without the need for DNA double-strand breaks.^25^^,^^26^^,^^27^ While this approach typically requires unique base-editor (BE)-guide RNA (gRNA) combinations to correct each mutation, the current diversity of BEs with a variety of protospacer-adjacent motif (PAM) compatibilities and Food and Drug Administration (FDA) receptivity to platform-technology approvals make this a promising strategy for the treatment of a broad range of genetic disorders.^28^ Given that many forms of PID and SCID stem from single pms, the base-editing approach holds promise as an ideal treatment strategy. Moreover, PIDs, including SCID, are ideal indications for the initial development of personalized gene-correction approaches given that these diseases can be attributed to pathogenic mutations in >500 different genes.^29^ Recent studies have demonstrated that base editing is suitable for HSCT through the reversion of sickle cell disease and X-linked SCID in mouse models,^30^^,^^31^ with promising clinical results of base editing of human HSPCs in patients as well^32^ (clinical trials NCT05456880, NCT06851767, and NCT06325709). However, the HSPCs in these studies were edited by electroporation, and the recipients were conditioned with either chemotherapy or IR—which carry toxicities to the transplanted cells and recipient tissues, respectively. For a treatment approach to be broadly applicable to all patients, it should be both safe and effective.

Virus-like particles (VLPs) offer an alternative to electroporation of HSPCs, and they have proven to safely and transiently deliver gene-editing reagents to various cell types.^33^^,^^34^^,^^35^^,^^36^^,^^37^^,^^38^^,^^39^^,^^40^^,^^41^ VLPs offer the transduction efficiency and tissue-targeting characteristics of retroviral delivery while circumventing the risk of viral genome integration by packaging ribonucleoprotein (RNP) cargo in place of viral genomic RNA. Furthermore, the transient expression of BEs or Cas9 RNPs as VLP cargo has been shown to limit off-target base editing.^42^^,^^43^^,^^44^ Our recently developed engineered VLPs (eVLPs) further enhance *ex vivo* and *in vivo* base editing with minimal off-target effects, offering a promising path to improve the safety and efficacy of HSPC gene-editing therapies, particularly by modulating the eVLP cellular tropism by utilizing different envelope glycoproteins.^45^ Hence, eVLPs represent a promising and versatile delivery modality to enable precision gene editing in HSPCs.

In addition to optimal correction of HSPCs, complementary ideal recipient conditioning is required, with current protocols for engraftment of gene-modified HSPCs still utilizing genotoxic chemotherapy conditioning, which can lead to serious toxicities. This has been highlighted in the recent HSPC base-editing clinical trial for sickle cell disease where a patient death was attributed to the use of busulfan chemotherapy conditioning.^32^ While immune suppression is not required in the setting of autologous hematopoietic stem cell (HSCs), we have previously shown that recipient HSCs limit engraftment of transplanted HSCs.^46^^,^^47^ This challenge could be addressed using monoclonal antibody (mAb)-based conditioning, as has been shown with αCD117 mAbs and antibody-drug conjugates (ADCs). This approach has exhibited promise in safely depleting recipient HSCs while enabling efficient engraftment of unmanipulated donor HSPCs in mice, non-human primates, and, more recently, clinical trials treating patients with SCID.^46^^,^^48^^,^^49^^,^^50^^,^^51^^,^^52^^,^^53^ However, it is unknown if this type of pre-treatment conditioning can be effectively combined with base-edited HSPCs.

In this study, we established a *Rag2*-SCID mouse model with a single pm, *Rag2* c.107G>A, to explore base editing in the context of SCID. We also developed delivery methods for HSPCs and report the *ex vivo* editing of mouse HSPCs using eVLPs containing BEs. These tools were then combined to investigate the correction of *Rag2*^*pm*^ HSPCs using eVLPs containing adenine BEs (ABEs). Importantly, we also combined this editing approach with mAb-based conditioning to circumvent the use of toxic conditioning. Two eVLP formulations containing SpCas9NG-ABE8e were tested to edit HSPCs from the *Rag2*^*pm*^ mice. *Rag2*^*pm*^ HSPCs transduced with the optimal eVLPs were transplanted into *Rag2*^*pm*^ mice using non-genotoxic αCD117-ADC conditioning. Immunophenotyping was subsequently performed, which demonstrated that even a very small number of edited HSPCs can alleviate SCID. This work demonstrates, as proof of principle, a safe and effective treatment approach for PIDs that are caused by single pms.

## Results

### Generation of a *Rag2* point-mutant mouse model

We aimed to develop a SCID mouse model lacking mature T or B cells that could sensitively test for efficacy of base editing. The *Rag2* gene was selected, given this is a frequent cause of SCID that results in complete absence of both mature T and mature B cells. The *Rag2* gene consists of three exons, featuring the ATG start codon and TAA stop codon within exon 3. The Rag2 protein comprises two critical domains: the N-terminal core region (amino acids 1–383) and the C-terminal plant homeodomain finger (PHD) (amino acids 384–527). The core region plays a pivotal role in DNA cleavage activity, interacting with Rag1 to form the RAG complex, which is essential for V(D)J recombination. Meanwhile, the PHD binds to histone H3 trimethylated on lysine 4 (H3-K4me3) and H3 peptide. This enables Rag2 to bind to regions of active chromatin, thereby augmenting the catalytic activity of the RAG complex.^54^^,^^55^ Research indicates that while the Rag2 C-terminal region is crucial for maintaining genomic stability, it is not essential for the recombination,^56^ as its suppression leads to only a slight reduction in the total number of B and T cells.^57^

**Figure 1:**
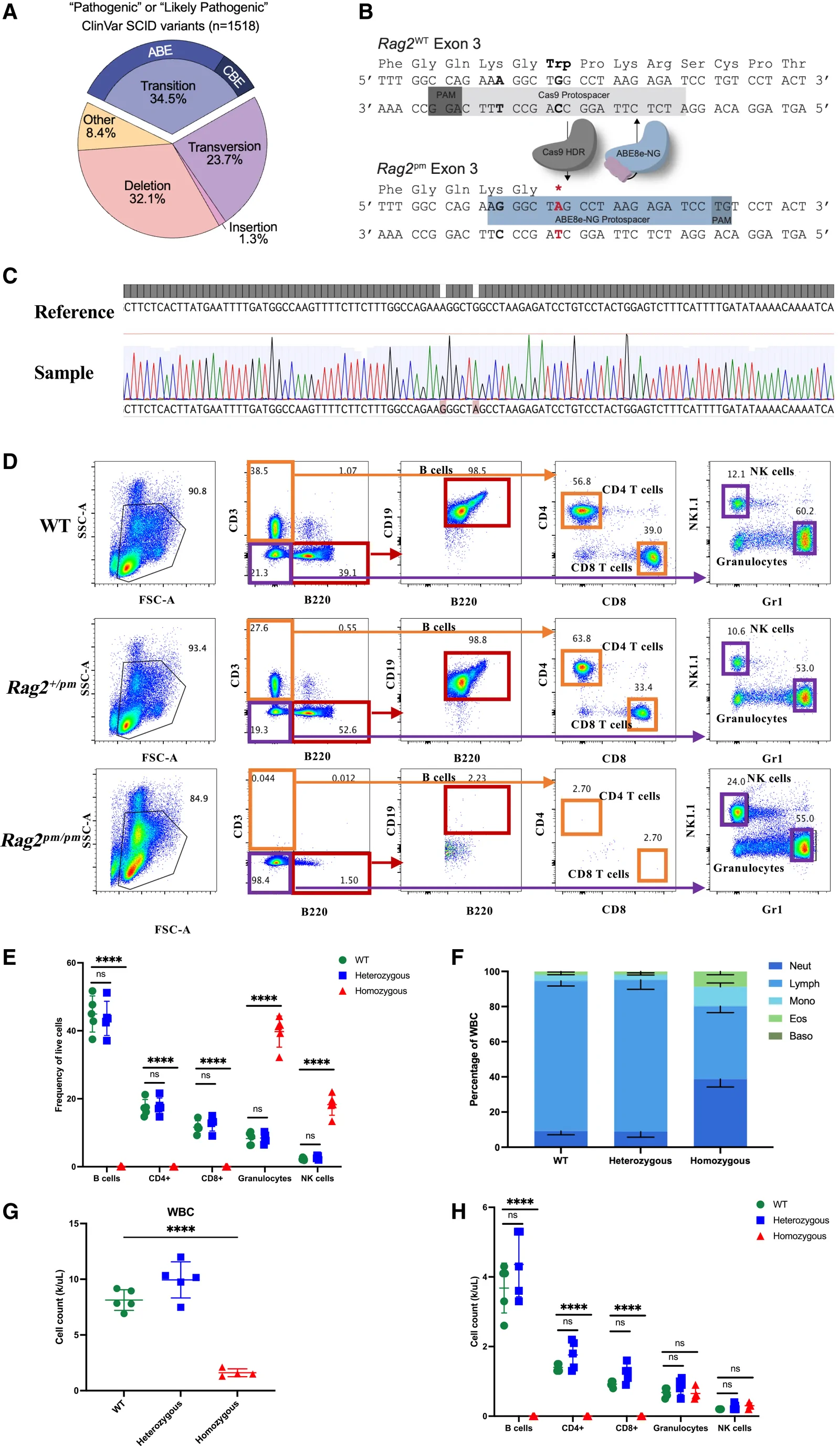
Generation of *Rag2* c.107G>A point mutant a mouse model (A) Distribution of SNVs reported on ClinVar that are associated with SCID and categorized as “pathogenic” or “likely pathogenic.” (B) Sequence of mutant *Rag2* allele containing the early stop codon candidate with the complementary guide RNA used to correct the mutation with SpABE8e-NG. Silent mutation in blue and target nucleotide in red introduced by HDR and restored to WT by SpNG-ABE. (C) Sanger sequencing shows the introduction of *Rag2* W36X and the synonymous mutation p.K34. (D and E) Flow cytometry analysis shows a lack of T and B cell production in *Rag2*^*pm/pm*^ mice compared with WT and *Rag2*^+/*pm*^ mice. Error bars: SD. CBCs show (F) low lymphocyte frequency and (G) low WBC counts in *Rag2*^*pm/pm*^ mice compared with WT and *Rag2*^+/*pm*^ mice. Error bars: SD. (H) *Rag2*^*pm/pm*^ mice exhibit normal NK cell and granulocyte counts comparable to those of WT and *Rag2*^+/*pm*^ mice. Error bars: SD.

Thus, to achieve a complete knockout, we screened the murine *Rag2* core sequence for specific codons, including TGG for tryptophan (W), AGA/CGA for arginine (R), and CAA/CAG for glutamine (Q), to introduce an in-frame stop codon that could potentially be reverted by ABE. ABE was chosen as it is theoretically capable of correcting 27% of pathogenic or likely pathogenic SCID-causing mutations cited on ClinVar, with an additional 7% of causal mutations being addressable by CBEs (Figure 1A). To demonstrate a method that could be used broadly for SCID single-nucleotide mutations—even those lacking ideal PAMs—we sought to introduce a challenging mutation that requires the non-canonical Cas9-NG. We identified eight potential candidates with a target nucleotide positioned 12–16 bases upstream of an NG PAM site (Q4X, W36X, Q52X, Q166X, R167X, W172X, R212X, and R349X). Among these, W36X, Q52X, and R212X had no potential bystander adenines within the ABE8e activity window (protospacer positions 3–10).^26^ We chose to induce the W36X mutation in the *Rag2* gene as it is the farthest from the PHD (Figure 1B). CRISPR-Cas9 homology-directed repair (HDR) machinery was utilized to create the C57BL/6NTac *Rag2* pm mouse model. A synonymous mutation, p.K34 (AAA to AAG), was introduced to prevent the binding and re-cutting of the sequence by the gRNA after HDR (Figure 1B). PCR genotyping followed by Sanger sequencing analysis was performed on the pups. Four founders were then backcrossed with C57BL/6NTac to establish the initial breeding pairs. Heterozygous mice were subsequently bred to generate the first homozygous “*Rag2*^*pm/pm*^” mouse (Figure 1C).

Flow cytometry analysis of peripheral blood (PB) from the SCID *Rag2*^*pm/pm*^ mice demonstrated a lack of B cells, CD4^+^ T cells, and CD8^+^ T cells (Figures 1D and 1E). Peripheral white blood cell (WBC) counts further revealed a lower lymphocyte frequency (Figure 1F). The distribution of cell types in WBCs is shown to highlight the reduced lymphocyte proportion in *Rag2*^*pm/pm*^ mice compared with wild-type (WT) and heterozygous *Rag2*^+/*pm*^ controls. However, the overall WBC count was significantly lower in *Rag2*^*pm/pm*^ mice (Figure 1G), primarily due to their severely diminished lymphocyte numbers (Figure 1H). As a result, the absolute numbers of granulocytes and natural killer (NK) cells in the *Rag2*^*pm/pm*^ mice are not increased compared with WT and heterozygous controls (Figure 1H). The elevated frequencies of granulocytes and NK cells observed by fluorescence-activated cell sorting (FACS) (Figure 1E) are relative, reflecting the absence of T and B cells in these *Rag2*^*pm/pm*^ mice.

### High editing efficiency of HSPCs transduced with SpCas9-ABE8e-eVLPs

Electroporation has been commonly used in previous studies to deliver nucleic acid cargoes to HSPCs.^30^^,^^31^ However, this causes cell injury and requires prolonged cell culturing for recovery, which can trigger HSPC differentiation. To address this, we compared electroporation to newly developed eVLPs for the correction of HSPCs. eVLPs are recognized for their high transduction efficiency and their ability to deliver RNPs, reducing the risk of off-target effects.^45^ Indeed, in head-to-head studies comparing *S. pyogenes* (Sp) Cas9-ABE8e RNP electroporation and eVLP-mediated delivery to mouse c-kit^+^ HSPCs, cell viability was higher with eVLPs (99.26%) compared with electroporation (77.55%) (*p* < 0.0001). Further, eVLP-transduced c-kit^+^ HSPCs showed significantly higher base editing efficiency (*p* = 0.0269) versus electroporated cells (Figures S1A and S1B).

One of the key advantages of VLPs is their modular surface glycoproteins, which allow customizable cellular targeting.^45^ BaEV-Rless is a modified version of the baboon endogenous retrovirus envelope glycoprotein (BaEV) in which the R peptide inhibiting cell-to-cell fusion was removed from the C terminus. BaEV-Rless-pseudotyped lentivirus has demonstrated higher transduction efficiency in HSCs compared with vesicular stomatitis virus G protein lentiviral vector (VSV-G-LV).^58^^,^^59^ Similarly, the VSV-G/BaEV-Rless co-pseudotype increases c-kit^+^ cell editing by 2.5-fold (Figure S1C). Hence, for subsequent *Rag2*^*pm*^ correction studies of mouse HSPCs, BaEV-Rless/VSV-G co-pseudotyped eVLPs were used.

**Figure 2:**
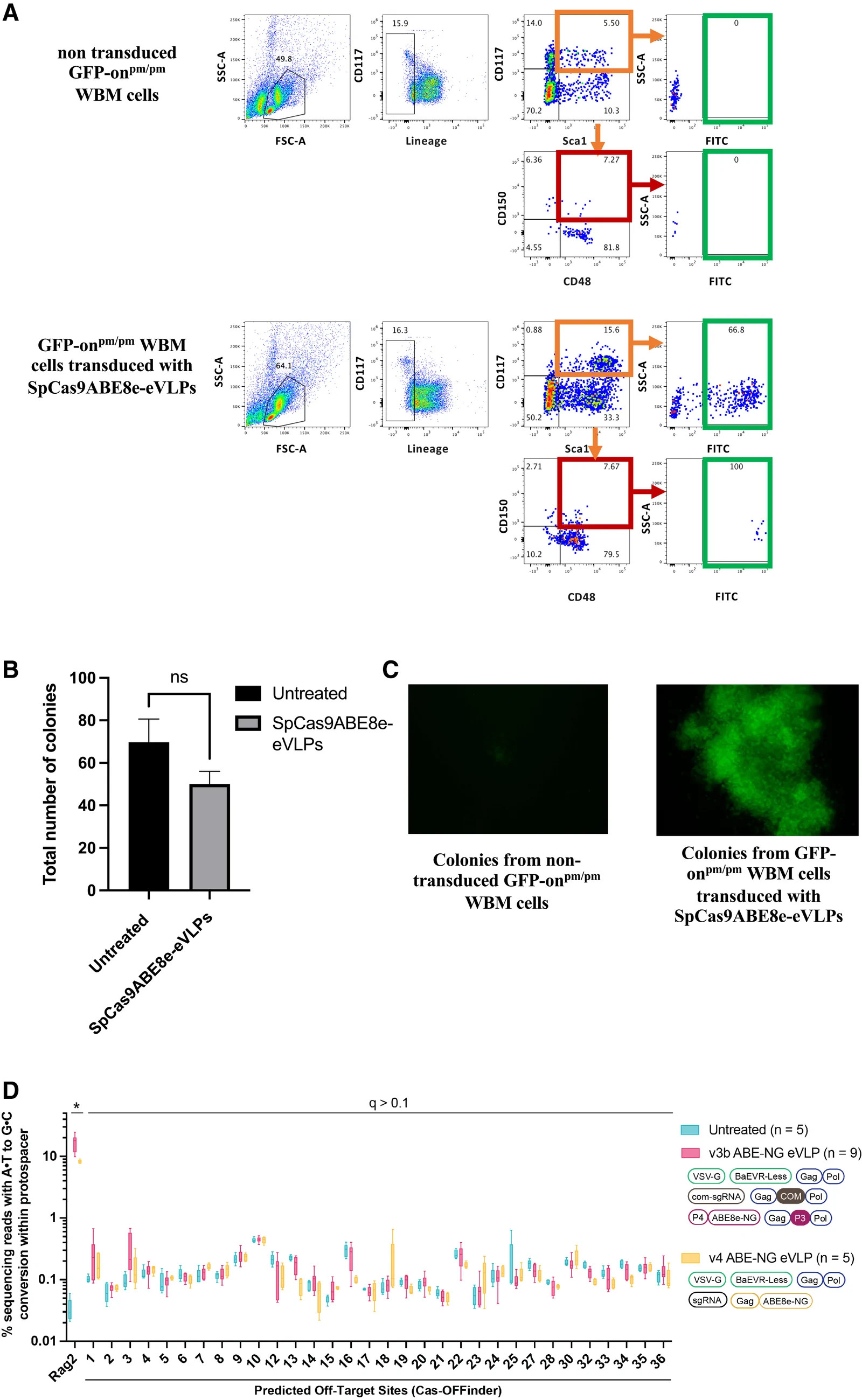
HSPC editing with BE-eVLPs (A) FACS plots showing EGFP expression in *GFP-on*^*pm/pm*^ HSCs (LSK CD150^+^CD48^−^) 48 h post-transduction with SpCas9ABE8e-eVLPs. (B) The differentiation potential of HSPCs, assessed by colony-forming cell assay, is not affected by eVLP transduction. Error bars: SD. (C) Fluorescence microscopy images show EGFP expression in colonies generated from *GFP-on*^*pm/pm*^ progenitor cells transduced with SpCas9ABE8e-eVLPs. (D) High-throughput sequencing (HTS) results show editing using the v3b formulation increases editing by 2-fold in *Rag2*^*pm/pm*^ HSPCs compared with v4 eVLPs with no significant off-target levels (*q* < 0.1) observed compared with *Rag2*^*pm/pm*^ untreated control cells. The on-target and top 36 off-target sites nominated by Cas-OFFinder that successfully amplified are plotted. Error bars: SD.

To further evaluate the transduction efficiency of BaEV-Rless/VSV-G co-pseudotyped eVLPs (v4) in mouse HSCs, we used the *GFP-on* mouse model, which harbors a premature termination codon within the EGFP gene that is restored by ABE8e delivery.^60^ Transduction of whole bone marrow (WBM) cells from *GFP-on*^*pm/pm*^ mice with BaEV-Rless/VSV-G co-pseudotyped SpCas9-ABE8e eVLPs led to complete restoration of EGFP expression in 100% of HSCs (Lin^−^Sca1^+^c-Kit^+^ [LSK] CD150^+^CD48^−^), as detected by flow cytometry (Figure 2A). Furthermore, a colony-forming cell (CFC) assay was performed on the cells, which confirmed that the HSPC colony potential remained unaffected by the eVLP transduction, with EGFP expression observed by microscopy (Figures 2B and 2C). These observations demonstrate the potential of eVLP delivery to target and edit phenotypic HSPCs within the mixed WBM—further expanding their utility in engraftment studies.

### *Ex vivo* correction of *Rag2*^*pm/pm*^ HSPCs

HSPCs obtained from the BM of *Rag2*^*pm/pm*^ mice were subsequently transduced using similar methods with v4 containing SpCas9NG-ABE8e. The transduction of *Rag2*^*pm/pm*^ HSPCs with eVLPs at 7.2E5 MOI resulted in an average correction rate of 8.3% 48 h post-transduction (Figure 2D). To increase the editing efficiency, we next modified the eVLP formulation. Specifically, it has been recently noted by our lab and others that using additional protein-protein and aptamer-protein interactions to load RNP cargo into eVLPs affords substantial improvement in eVLP-mediated base and prime editing efficiencies *ex vivo* and *in vivo*.^38^^,^^43^^,^^61^^,^^62^ We utilized the COM-com protein-RNA aptamer interaction by installing a com aptamer in stem loop II of the single guide RNA (sgRNA) scaffold (com-sgRNA; supplemental information) and the COM protein into the Gag-Pol polyprotein (Gag-COM-Pol) to recruit additional sgRNAs to eVLPs.^34^ We also employed the P3-P4 coiled-coil peptide interaction by fusing P4 to SpABE8e-NG (P4-SpABE8e-NG; supplemental information) and P3 to the Gag-Pol polyprotein (Gag-P3-Pol) to assist in BE protein loading.^62^ This eVLP formulation is hereafter denoted v3b.^62^ Together, these additional binding modes improved overall editing 2-fold to 16.4% using identical transduction conditions (Figure 2D). This gain in editing efficiency is likely due to more efficient encapsulation of editor components within eVLPs offered by the additional binding modes for both the sgRNA alone and the ABE-sgRNA RNP complex.

Off-target sites for SpABE8e-NG were identified using Cas-OFFinder, and the 36 protospacers with the highest MIT score and an adenine in the defined editing window—ranked by number of base mismatches in the protospacer and their proximity to the 5′ end of the protospacer sequence—were nominated. Only 1 of the 36 evaluated off-target loci is exonic, with OT9 located in the first exon of *Ankrd50*, a protein involved in endosome recycling. We assessed the editing efficiency at these 36 loci by targeted amplicon sequencing and did not find any statistically significant off-target editing in v4 or v3b ABE8e-NG-eVLP-treated *Rag2*^*pm/pm*^ HSPCs treated *ex vivo* (Figure 2D). The specific off-target genomic loci identified are detailed in Table S1.

### Engraftment of v3b ABE8e-NG-eVLP-transduced HSPCs in *Rag2*^*pm/pm*^ mice

**Figure 3:**
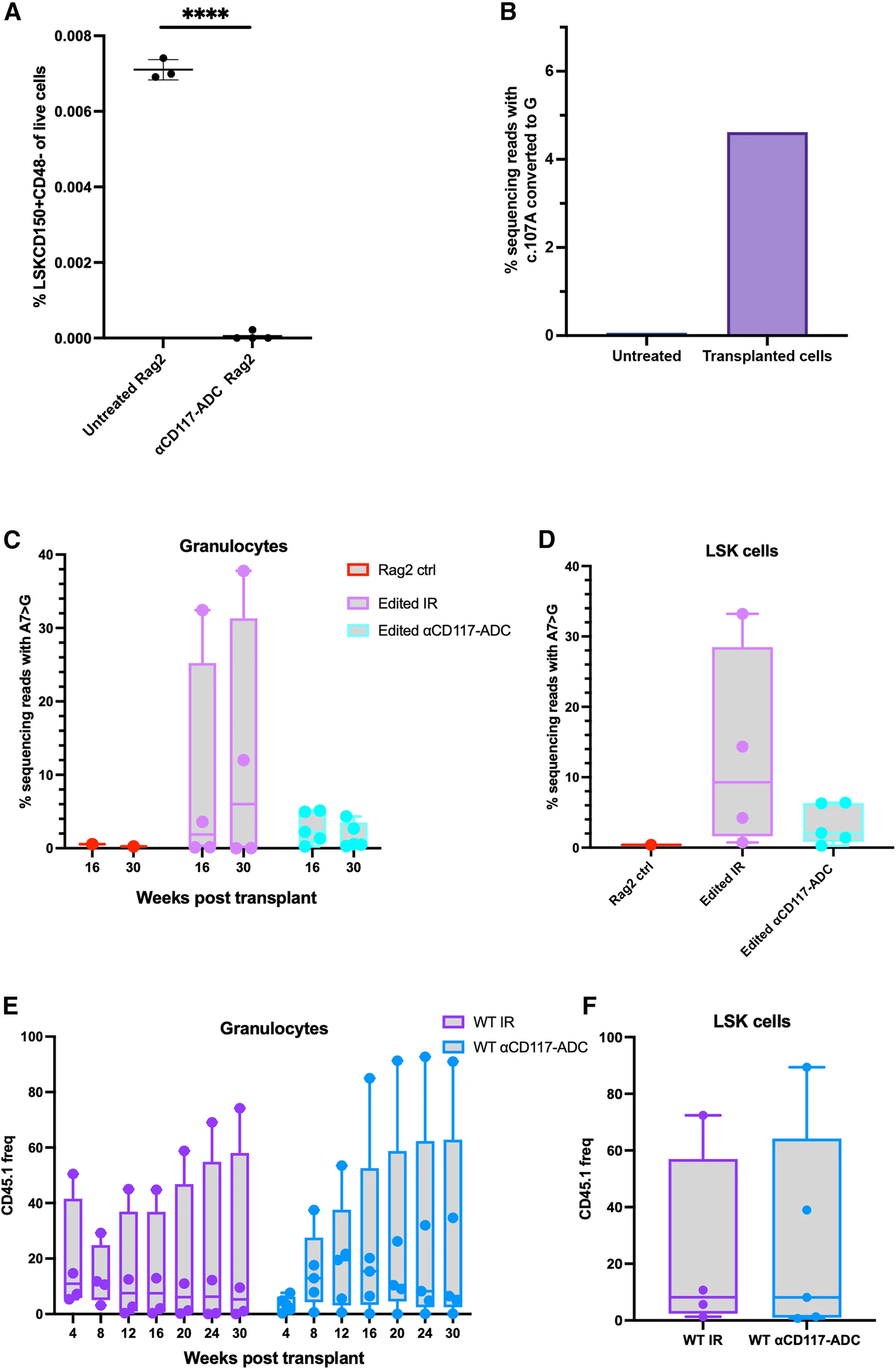
CD117-ADC treatment and engraftment in *Rag2*^*pm/pm*^ mice post-transplantation (A) Depletion of HSCs (LSK CD150^+^CD48^−^) in the bone marrow of *Rag2*^*pm/pm*^ mice treated with αCD117-ADC. Error bars: SD. (B) ∼5% of c-kit^+^ HSPCs transplanted into mice were corrected. Correction frequency in (C) granulocytes and (D) c-kit^+^ HSPCs from *Rag2*^*pm/pm*^ mice transplanted with SpABE8e-NG-eVLP-transduced HSPCs as assessed by HTS. (E) Donor granulocyte and (F) LSK HSPC chimerism in *Rag2*^*pm/pm*^ mice transplanted with similarly cultured WT CD45.1 HSPCs as evaluated by flow cytometry analysis. Boxplots show the median, range, and quartiles.

After optimizing *Rag2*^*pm*^ HSPC editing *ex vivo*, we investigated whether eVLP-transduced cells can engraft and if a low number of corrected HSPCs can revert the *Rag2* SCID phenotype. *Rag2*^*pm/pm*^ mice were first conditioned with IR or αCD117-ADC.^49^^,^^63^ Eight days after conditioning, a BM aspirate was performed on a subset of the Ab-conditioned mice to assess for HSC depletion. Assessment by flow cytometry showed near-complete depletion of HSCs in treated mice compared with untreated controls (Figure 3A).

WBM cells were next isolated from the *Rag2*^*pm/pm*^ mice, c-Kit^+^ enriched to mimic clinical CD34^+^ enrichment, and transduced with v3b ABE8e-NG-eVLPs at 7.2E5 MOI. To avoid *ex vivo* differentiation of the HSPCs, they were transplanted into *Rag2*^*pm/pm*^ mice 24 h post-transduction. In parallel, c-Kit^+^ HSPCs from WT (CD45.1) donors that were similarly cultured were also tested. *Rag2*^*pm/pm*^ mice were transplanted with only 100,000 v3b ABE8e-NG-eVLP-transduced *Rag2*^*pm/pm*^ HSPCs or 100,000 WT HSPCs. Groups were labeled as follows: edited IR (*n* = 5), edited αCD117-ADC (*n* = 5), WT IR (*n* = 5), and WT αCD117-ADC (*n* = 5). High-throughput sequencing (HTS) was also performed in parallel on a fraction of the transduced cells that were transplanted, which showed that ∼5% of the alleles were corrected (Figure 3B).

To determine the engraftment of eVLP-transduced and WT cells, HTS was performed on sorted granulocytes at 16 and 30 weeks, and percentage donor CD45.1 frequency was assessed in granulocytes by flow cytometry every 4 weeks, respectively. Complete blood counts (CBCs) and PB flow cytometry analyses were performed every 4 weeks to evaluate PB lineage composition. Thirty weeks post-transplantation, we determined edited and WT HSPC engraftment, long-term lymphocyte regeneration, and B cell progenitor composition. HTS results showed that six out of nine mice transplanted with transduced HSPCs had corrected granulocytes in PB with varying degrees of editing up to 32% at 16 weeks post-transplantation (Figure 3C). As expected at 30 weeks post-transplant, the percentage of corrected HSPCs in the BM mirrored the PB granulocyte HTS results (Figure 3D). Notably, regardless of conditioning method, we observed significant engraftment variability across the mice, which was also true of mice transplanted with the same number of WT cultured cells (Figures 3C–3F), likely due to the low number of engraftable HSPCs that were transplanted post-culture.^31^^,^^64^

### v3b ABE8e-NG-eVLP-transduced HSPCs revert the SCID phenotype

**Figure 4:**
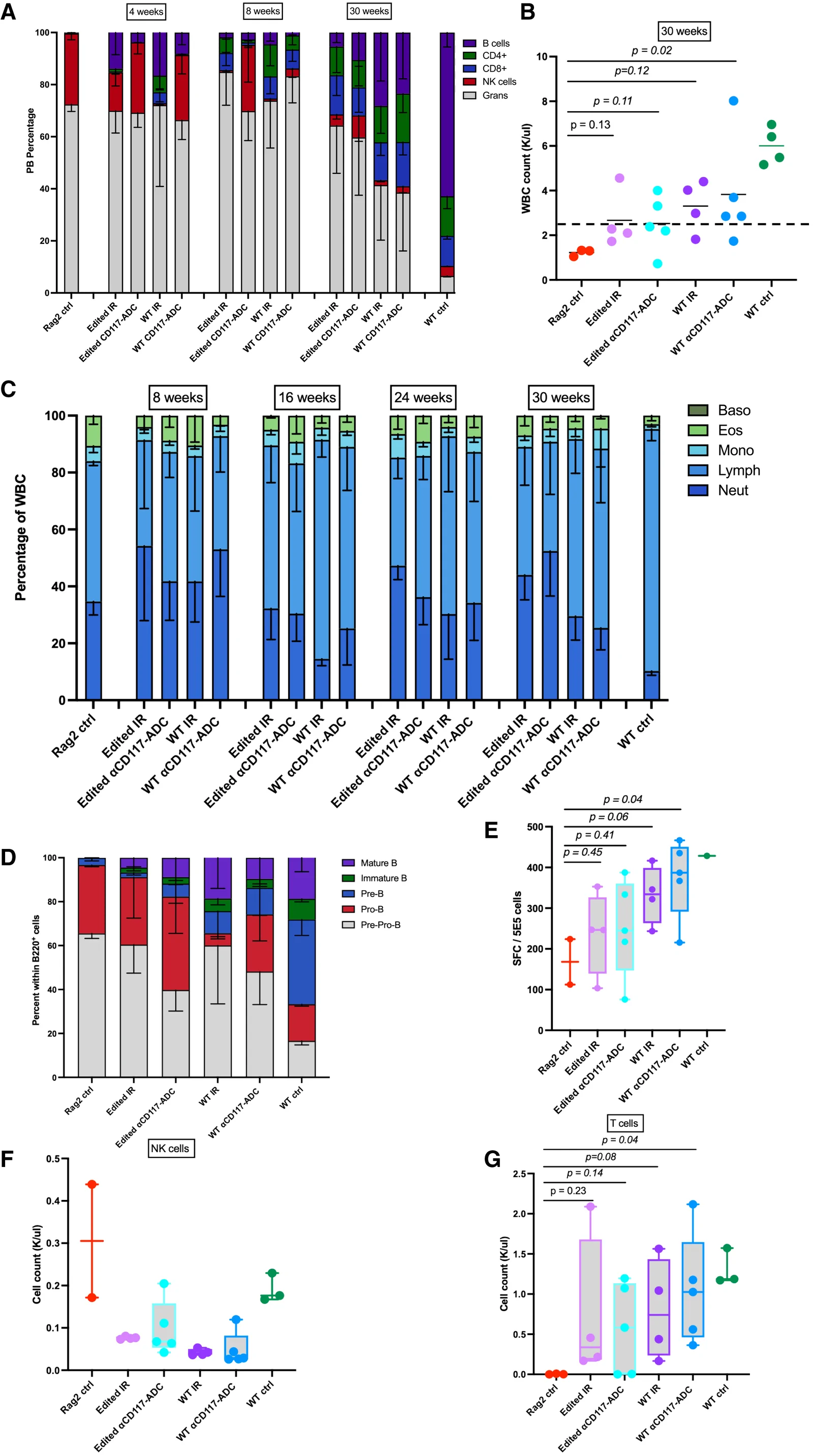
Immune reconstitution in transplanted *Rag2*^*pm/pm*^ mice (A) T and B cell production was observed in transplanted mice beginning at 4 weeks and continuing until 30 weeks post-transplantation as assessed by flow cytometry. Error bars: SD. (B) At 30 weeks post-transplantation, the WBC count was higher in nearly all transplanted mice compared with the *Rag2*^*pm/pm*^ untreated control group. The dashed line indicates the WBC count observed in the *Rag2* control group. (C) Lymphoid lineage composition in mice from peripheral blood at 8, 16, 24, and 30 weeks post-transplantation. Error bars: SD. (D) Percentage composition of B cell progenitors in the bone marrow at 30 weeks post-transplantation. Error bars: SD. (E) ELISpot assay results from transplanted mice. Each mouse was tested in duplicate. The average number of spots from the unstimulated sample was subtracted from samples stimulated by PMA and ionomycin cocktail. SFC, spot-forming cells. The total (F) NK cell and (G) T cell counts in PB were calculated by multiplying their frequency obtained from flow cytometry analysis by WBC obtained from CBC. Boxplots show the median, range, and quartiles.

Flow cytometry analysis of the PB revealed that B cell production began within the first month post-transplantation across all groups and persisted until the seventh month (Figure 4A). T cell regeneration also began at 4 weeks in mice transplanted with WT HSPCs and at 8 weeks in some mice from the groups transplanted with gene-edited HSPCs, with a gradual increase over time (Figures 4A and S2A). T and B cell regeneration was tracked using flow cytometry and CBCs, which allowed for calculating the total number of cells in the PB, further confirming gradual increases over time (Figure S2B).

To verify that the newly produced T and B cells originated solely from the base-edited HSPCs, we sorted these from *Rag2*^*pm/pm*^ mice that had been transplanted with v3b-transduced HSPCs 16 weeks post-transplantation and performed HTS. The HTS results indicated that 50% of the T and B cell alleles had been corrected, suggesting that the correction occurred monoallelically in the edited HSPCs (Figure S2C). Furthermore, all alleles contained the silent mutation p.K34 (AAA to AAG), confirming that the sorted cells were derived exclusively from the edited *Rag2*^*pm/pm*^ cells and ruling out any contamination with WT cells at the time of transplantation. CBC analysis showed that WBC counts in a majority of the transplanted mice were higher than those of *Rag2*^*pm/pm*^ untreated controls (Figure 4B and 4C).

Additionally, to evaluate the correction of B cell differentiation in the BM, we conducted flow cytometry analysis on BM cells 30 weeks after transplantation. While B cell progenitors in *Rag2*^*pm/pm*^ mice were arrested at an early stage of differentiation, the mice treated with base-edited HSPCs had BM progenitors that progressed through all stages of differentiation similar to B cell progenitors in WT mice (Figures 4D and S3). Notably, in mice with ∼1.5% or greater corrected or WT HSPCs, robust T cell (Figures S4A and S4B) and B cell (Figures S4C and S4D) production was observed. To verify the robustness of these findings, these experiments were repeated in a separate replicate experiment showing similar results (Figure S5).

Last, to assess the functionality of the base-edited T cells, we conducted an ELISpot assay on blood cells isolated from the spleen of transplanted mice. This test confirmed that the T cells could produce IFN-γ in response to stimulation with a PMA (phorbol 12-myristate 13-acetate) and ionomycin cocktail (Figure 4E). As the murine spleen contains also NK cells, which can be activated by PMA and ionomycin,^65^ we measured the total number of NK cells and T cells in the PB using flow cytometry and CBCs. The absolute number of NK cells in the transplanted mice was comparable to that in *Rag2*^*pm/pm*^ controls (Figure 4F). This indicates that the increased number of spots observed in the transplanted mice samples could be attributed to the newly generated T cells (Figure 4G).

## Discussion

Clinical studies have demonstrated that correcting even a small fraction of HSPCs can result in significant clinical improvements or even lead to disease cure in SCID patients.^66^ Given that SCID can be caused by single pms, base editing offers a promising treatment approach, as highlighted in a recent study in SCID mice, though this work utilized electroporation and genotoxic conditioning.^31^ Moreover, the clinical development of BEs for other diseases^32^ and the recent FDA designation for platform therapies may make this approach feasible for patients with various SCID mutations in the future. However, for this approach to be applied broadly to patients it would need to be safe and effective. Here, we aimed to assess whether eVLPs could serve as a viable method for delivering BEs to HSPCs with a goal to combine BE-eVLP HSPC transduction with αCD117-ADC conditioning, a safer and effective alternative in SCID therapy. Using BM cells from an EGFP point-mutant reporter mouse model, we demonstrated that BEs delivered via eVLPs could successfully transduce and edit HSPCs without impairing their differentiation potential. We next developed a *Rag2*^*pm/pm*^ mouse model lacking T and B cell production and harboring a pm that is correctable with SpCas9NG-ABE8e and further optimized the eVLP formulation to enhance the *ex vivo* correction of *Rag2*^*pm*^ HSPCs.

Mice were subsequently conditioned using either IR or αCD117-ADC and were transplanted with either WT or BE-eVLP-transduced HSPCs. While HTS revealed that, on average, only ∼5% of HSPC alleles were successfully edited, notably, the corrected HSPCs enabled robust and long-term donor cell engraftment, lymphocyte regeneration over 30 weeks, and reversal of the SCID phenotype in almost all mice in both irradiated and αCD117-ADC-conditioned settings. Specifically, in mice with ∼1.5% or greater editing or WT engraftment, robust B cell and T cell production was observed. In these mice, B cell progenitors progressed through all differentiation stages, and newly generated T cells were functionally active, producing IFN-γ in response to stimulation. However, considerable mouse-to-mouse variability was detected in both conditioned settings, likely owing to the low number of HSCs that were transduced and transplanted. This suggests that even low-efficiency editing with low HSC number transplantation is sufficient to cure SCID—hence, this could be an ideal indication for development of personalized BEs, as obtaining high-efficiency editing is not required and SCID is known to be caused by diverse mutations in >15 genes.^67^

These findings highlight the effectiveness of BEs in correcting SCID mutations, reinforcing their potential as a clinical tool for treating this disease. We also demonstrate that BaEV-Rless/VSV-G co-pseudotyped eVLPs are an efficient method for delivering editing tools to mouse HSPCs. More significantly, this study shows that combining HSPC editing using BE-eVLPs with a safe conditioning strategy, such as αCD117-ADC mAbs, is feasible. Further, there was a trend toward improved immune reconstitution with αCD117-ADC over IR, which may be due to the αCD117-ADC causing less recipient tissue injury. This study provides a strong rationale for advancing autologous base-edited HSCT protocols with non-genotoxic conditioning in treatment of SCID and other diseases.^68^

Our results demonstrate a safer alternative to lentiviral gene therapy, which poses the risk of insertional mutagenesis that may lead to oncogenesis. By utilizing eVLPs to deliver BE-RNPs, we further minimize the duration of exposure to the editing tools, thereby substantially reducing the chance of off-target editing.^45^ Further, as multiple αCD117 mAbs are being developed by various groups, with the first clinically available αCD117 mAb conditioning agent having already been tested in SCID patients with reported early safety and efficacy,^52^^,^^53^ these finding may be translatable in the near term. Though development of the first clinical αCD117-ADC was halted due to a study patient death, this was conclusively attributed to the drug, and additional naked mAbs, combination mAb approaches, and other αCD117-ADCs are also in development. These αCD117 mAb conditioning approaches may be both safe and well tolerated, facilitating successful donor cell engraftment without the severe side effects typically associated with conventional conditioning regimens. Considering these advancements, our study takes on particular significance. Our findings emphasize the critical opportunity for using αCD117 mAbs as toxicity-free conditioning methods that can ensure persistent HSPC engraftment even if low editing efficiency or limited HSPC numbers are utilized.

In addition to enabling improved HSPC viability over electroporation, the use of eVLPs in this work enables potential utility of this editing approach for *in vivo* correction of SCID, as eVLPs are being explored for *in vivo* applications in other organ systems and disease settings. Notably, SCID may be an ideal disease model for testing *in vivo* HSPC editing because only a modest level of correction is required for disease correction. While *in vivo* editing remains outside the scope of the reported work, it holds significant promise to further ease treatment, as it could eliminate the need for mobilization, apheresis, *ex vivo* HSPC graft manufacturing, and conditioning altogether while enabling enhanced immune recovery and vastly improving the scalability of autologous HSCT.

Although our work was performed in a specific representative point-mutant mouse model, it provides proof of concept of a safe and effective treatment approach to SCID. For clinical translation, personalized BEs would need to be created for each patient-specific mutation, which can also occur in a wide range of SCID-causing genes. Development of personalized editors by several groups is underway, and it is predicted that ∼34.5% of known SCID-causing mutations are transition mutations that can be reverted using BEs (Figure 1A). For the remaining mutations, prime editors—which are also compatible with eVLP delivery—and other genome-editing tools could likely be utilized, though this is outside the scope of the work presented here. An important consideration shown through this work is that even low-level mutation correction with relatively inefficient BE variants can still lead to safe and effective disease correction. While optimization will need to be performed for each patient-specific BE prior to clinical use to maximize on-target and decrease off-target editing, even for patients with challenging-to-correct mutations with only moderate editing efficiency, our work shows that this approach could still result in disease correction.

In summary, our findings confirm the following key points: (1) HSPCs can be successfully edited using BEs delivered via eVLPs; (2) HSPCs transduced by eVLPs are capable of engrafting and effectively correcting the SCID phenotype; (3) a minimal number of corrected HSPCs is sufficient to reverse the SCID phenotype; and (4) the use of αCD117-ADC mAbs as a conditioning agent facilitates engraftment of BE-eVLP-transduced HSPCs with efficiency similar to that of IR.

## Materials and methods

### Mice

The C57BL/6-CD45.1STEM mice were generously provided by Prof. David Scadden from Harvard University and served as donors for transplantation experiments. For all studies, mice ages 12–16 weeks were used. All experiments were conducted in accordance with the guidelines and approvals of Stanford University Institutional Animal Care and Use Committee (IACUC).

### Generation of *Rag2* c.107G>A mouse model

The Rag2 W36X mouse model was generated by microinjection of SpCas9 protein, a targeted gRNA (5′-ATCTCTTAGGCCAGCCTTTC-3′), and a single-strand DNA template containing the Rag2 W36X mutation and a silent mutation, p.K34 (AAA to AAG), into fertilized eggs of WT C57BL/6NTac mice. Following the backcrossing of four founder animals, seven F1 animals were generated. The microinjection, the founders, and the F1 animals’ genotyping were done by the Cyagen Biosciences Company. Subsequently, F1 mice were backcrossed to C57BL/6N to set the first breeding pairs. *Rag2*^+/*pm*^ animals were intercrossed to obtain *Rag2* homozygous mutants (*Rag2*^*pm/pm*^). The pups were genotyped using the following primer followed by Sanger sequencing: donor oligo (the silent mutation is highlighted in bold font and the mutation is underlined), AATTCAACCAGGCTTCTCACTTATGAATTTTGATGGCCAAGTTTTCTTCTTTGGCCAGAA**G**GGCTAGCCTAAGAGATCCTGTCCTACTGGAGTCTTTCATTTTGATATAAAACAAAATCATCTCAAA.

### Mice genotyping

Genomic DNA was extracted from ear clippings using the NucleoSpin Tissue kit (Macherey-Nagel). *Rag2* genotyping was conducted using the following primers: *Rag2* F (5′-CAAGCTGCTGCCACAATAAA-3′) and *Rag2* R (5′-TGCTGCCTTTGTATGAGCAA-3′). The PCR products were subsequently subjected to Sanger sequencing (Quintara Bio), and the data were analyzed using the Benchling platform to confirm the induction of the *Rag2* pm.

### CBCs

Hematology analysis was conducted using the Sysmex XN-1000V hematology analyzer system. CBC measurements were obtained from WT, heterozygous, and homozygous *Rag2* pm mice at 12 weeks of age to characterize the mouse model. CBC measurements were collected from WT, homozygous, and treated mice every 4 weeks post-transplantation to evaluate phenotype reversion in homozygous mice.

### BE-eVLP production and purification

BE-eVLPs were produced by transfection of Gesicle producer 293T cells as previously described.^45^^,^^62^ Gesicle cells were maintained in DMEM + GlutaMAX (Life Technologies) supplemented with 1% (v/v) fetal bovine serum and seeded in T75 flasks (Corning, Glendale, AZ) at a density of 5E6 cells per flask 1 day prior to jetPRIME transfection (Polyplus, Berkeley, CA) with a mixture of plasmids according to the manufacturer’s protocols. To produce v4 eVLPs, a mixture of plasmids expressing VSV-G (400 ng), BaEVRLess (700 ng), MMLVgag-pro-pol (3,375 ng), MMLVgag-3×NES-ABE8e-NG (1,125 ng), and sgRNA 1 (4,400 ng) was diluted in jetPRIME buffer to 500 mL, complexed with 20 mL of jetPRIME transfectant, and administered to each T75 flask. To produce v3b eVLPs, plasmids expressing VSV-G (400 ng), BaEVRLess (700 ng), MMLVgag-pro-pol (2,813 ng), Gag-COM-Pol (2,000 ng), Gag-P3-Pol (422 ng), P4-SpABE8e-NG (422 ng), and com-sgRNA (4,400 ng) were co-transfected into each T75 flask. Plasmids were prepared for transfection using the PlasmidPlus Maxi Kit (Qiagen). Forty to forty-eight hours post-transfection, the producer cell supernatant was harvested and centrifuged for 5 min at 500 × *g* to remove cell debris. The eVLP-containing supernatant was filtered through a 0.45-mm polyvinylidene fluoride (PVDF) filter (Merck, Darmstadt, Germany). The filtered supernatant was concentrated 2,000- to 3,000-fold by ultracentrifugation using a 20% (w/v) sucrose in a PBS cushion,^45^^,^^62^^,^^69^^,^^70^ which was gently layered under eVLP-containing supernatant. Ultracentrifugation was performed at 26,000 rpm for 2 h at 4°C using an SW28 rotor in an Optima XPN100 ultracentrifuge (Beckman Coulter, Brea, CA). Following ultracentrifugation, BE-eVLP-enriched pellets were re-suspended in cold PBS (pH 7.4) and centrifuged at 1,000 × *g* for 5 min to remove debris, and the supernatant was stored at −80°C.

### *GFP*^*pm/pm*^ WBM cell editing and assessment

*GFP*^*pm/pm*^ WBM cells were obtained by BM aspiration of mice. Red blood cells (RBCs) were removed with RBC lysis buffer (eBioscience, San Diego, CA). Cells were then re-suspended in and were cultured in HSPC expansion medium: F12 (Gibco, Grand Island, NY), 0.1% Poly(vinyl alcohol) (Sigma-Aldrich, St. Louis, MO), 1% P/S/glutamine (Gibco, Grand Island, NY), 1% Insulin-Transferrin-Selenium-Ethanolamine (Gibco, Grand Island, NY), 100 ng/mL mouse thrombopoietin (mTPO) (Peprotech, Cranbury, NJ), and 10 ng/mL stem cell factor (SCF) (Peprotech, Cranbury, NJ).^71^^,^^72^ The cells were transduced with SpABE8e BaEVRless-VSVG co-pseudotyped eVLPs at 2.7E5 MOI. Forty-eight hours later, EGFP expression was determined by FACS, and HSPC differentiation potential was assessed by CFC assay.

### CFC assay

eVLP-transduced GFP^pm/pm^ WBM cells were plated in triplicate in Methocult M3434 medium (StemCell Technologies, Vancouver, BC, Canada) 48 h post-transduction. Colonies were scanned and analyzed using STEMvision (StemCell Technologies, Seattle, WA). EGFP expression in these colonies was assessed by microscopy imaging using the Echo confocal microscope (BICO, San Diego, CA).

### *Ex vivo* editing of *Rag2*^*pm/pm*^ HSPCs

BM cells were harvested from *Rag2*^*pm/pm*^ mice, and single-cell suspensions were obtained by crushing the spine and the lower-extremity bones. After RBC removal with RBC lysis buffer, BM cells were enriched using mouse CD117 MicroBeads (Miltenyi Biotec, San Jose, CA) according to the manufacturer’s instructions. Enriched cells were counted using Muse Count & Viability Kit (Millipore Sigma, Burlington, MA). c-kit^+^ HSPCs were then re-suspended at 1E6 cells/mL in Stemspan SFEM medium supplemented with 1% glutamine, 50 ng/mL mTPO, 100 ng/mL SCF, 20 ng/mL IL-3, 100 ng/mL FLT3 ligand, and 8 μM Cyclosporin H (CsH) (Sigma-Aldrich). All cytokines were purchased from PeproTech. Cells were seeded in 96-well plates at 1E5 cells/well confluence. eVLPs were thawed on ice and centrifuged at 4°C at 17,000 × *g* for 5 min and then added to the cells at 7.2E5 MOI. Forty-eight hours post-transduction, genomic DNA was isolated with the NucleoSpin Tissue kit (Macherey-Nagel, Austin, TX) according to the manufacturer’s instructions, and HTS was performed to assess editing.

To compare electroporation with eVLP-mediated editing, c-kit^+^ cells were isolated as described above. Cells were then either transduced with eVLPs at 7.2E5 MOI or electroporated using a Lonza electroporator with program DS130. For electroporation, 20 million cells/mL were re-suspended in 20 μL P3 buffer. The RNP complex was generated by incubating 80 pmol ABE8e-NG BE with 280 pmol of the *Rag2*-targeting sgRNA for 20 min at room temperature. Post-electroporation, cells were cultured overnight in 24-well plates at 4E5 cells per well in the medium described above, excluding CsH. After 24 h, the viability of both electroporated and eVLP-transduced cells was assessed by FACS, and editing efficiency was measured by HTS.

### HTS of genomic DNA

Genomic DNA was isolated with the NucleoSpin Tissue Kit. Five to fifty nanograms was used as input for the first of two PCRs. Genomic loci were amplified in PCR1 using NEBNext polymerase (New England Biolabs, Ipswich, MA). PCR1 forward and reverse primers used for the *Rag2* locus and the 36 off-target loci identified using Cas-OFFinder are detailed in Table 2. PCR1 was performed as follows: 98°C for 3 min; 25–35 cycles of 98°C for 15 s, 1°C for 20 s, and 72°C for 30 s; and 72°C for 2 min. PCR1 products were confirmed on a 1% agarose gel. One microliter of PCR1 was used as input for PCR2 to install Illumina barcodes. PCR2 used nine cycles of amplification using NEBNext DNA polymerase (New England Biolabs, Ipswich, MA). Following PCR2, samples were pooled and gel purified in a 1% agarose gel using a Qiaquick Gel Extraction Kit (Qiagen, Germantown, MD). Library concentration was quantified using a Qubit High-Sensitivity Assay Kit (Thermo Fisher, Houston, TX). Samples were sequenced on an Illumina MiSeq instrument (paired-end read, read 1, 220–280 cycles; read 2, 0 cycles) using an Illumina MiSeq 300 v.2 Kit (Illumina, San Diego, CA).

### HTS data analysis

Sequencing reads were demultiplexed using the MiSeq Reporter software (Illumina, San Diego, CA) and were analyzed using CRISPResso2 under batch analysis mode.^54^ Reads were filtered by minimum average Phred score (*Q* > 30) prior to analysis. The following window parameters were used: -w 20 -wc 10. Base editing efficiencies are reported as the percentage of sequencing reads containing a given base conversion at the specified position. Prism 9 (GraphPad) was used to generate bar plots of sequencing data.

### Off-target assessment in *Rag2*^*pm/pm*^ HSPCs transduced with SpCas9NG-ABE8e eVLPs

The Cas-OFFinder algorithm^73^ was used to search for protospacer sequences highly similar to the *Rag2*^*pm*^ ABE protospacer (AGGGCTAGCCTAAGAGATCC) with a 5′-NG-3′ PAM and an adenine in positions 1–11 within the off-target protospacer within the *Mus musculus* (mm10) genome. Candidate protospacers with four or fewer mismatches and a maximum DNA-RNA bulge size of 1 were ranked by computing the MIT score.^74^ This yielded a list of 36 protospacers with the highest MIT score and an adenine in the defined editing window. HTS was then performed on v3b and v4 ABE8e-NG-eVLP-treated and control *Rag2*^*pm/pm*^ HSPCs, and Welch’s *t* test was conducted to report *p* values between the observed editing efficiencies in these two groups.

### Transplantation into *Rag2*^*pm/pm*^ mice

HSPCs from *Rag2*^*pm/pm*^ and C57BL/6N-CD45.1^STEM^ were obtained as described above. *Rag2*^*pm/pm*^ c-kit^+^ cells were transduced as described above. Twenty-four hours post-culture, cells were pooled, washed, and re-suspended in PBS at 1E6 cells/mL. One hundred microliters of WT or *Rag2*^*pm/pm*^ transduced cells was transplanted intravenously via retro-orbital injection into *Rag2* homozygous mice. Transplanted mice were irradiated (800 cGy delivered in two doses, with the second on the transplantation day) or conditioned with αCD117-ADC 8 days prior to transplantation. αCD117-ADC (clone 2B8-Biotin, BioLegend, San Diego, CA; Streptavidin-ZAP, Advanced Targeting Systems, Carlsbad, CA) was prepared as described previously and administered at 1.5 mg/kg via intravenous injection 8 days before transplantation.^49^ One day prior to transplantation, a BM aspirate was collected from four of the ten Ab-conditioned mice to evaluate HSC depletion through flow cytometry analysis.

### Postmortem examination of transplanted Rag mice

To evaluate chimerism, PB was drawn from the retro-orbital sinus into heparinized tubes every 4 weeks following transplantation. Engraftment was analyzed via flow cytometry using mouse anti-CD45.1-PB and mouse anti-CD45.2-BV605 antibodies (both from BioLegend, San Diego, CA). Mice were euthanized for necropsy 30 weeks post-transplantation. BM cells were flushed from the femur with PBS and analyzed via FACS to assess B cell progenitor differentiation in all transplanted mice and BM chimerism in those transplanted with WT HSPCs. A detailed list of antibodies for flow cytometry analysis is provided in Table 3. LSK cells were sorted from mice transplanted with v3b-transduced HSPCs to determine chimerism by HTS. Spleens were collected for ELISpot analysis.

### ELISpot

Murine spleens were harvested from experimental mice, homogenized, and passed through a 70-μm cell strainer (Corning, 431751) in RPMI 1640 medium (Gibco, 11875-093). RBCs were lysed with 1× Red Blood Cell Lysis Buffer (Biolegend, 420302). Cells were centrifuged for 5 min at 400 × *g* at 10°C and re-suspended in RPMI 1640 supplemented with 10% FBS (Sigma-Aldrich, 12306C), 1% penicillin-streptomycin (Gibco, 15140-122), 1% L-glutamine (Sigma-Aldrich, G7513), and 0.1% 2-mercaptoethanol (Sigma, M-7522). Cells were filtered through a 70-μm cell strainer and counted using the Muse Cell Analyzer (Muse, 0500-3115). Cells (5E5/100 μL) were plated in each well. The cells were incubated with 1× PMA ionomycin (Thermo Fisher, 00-4970-93) for 48 h at 37°C. ELISpot assay (R&D Systems, EL485) was performed according to the manufacturer’s instructions. Plates were read using an ImmunoSpot Analyzer (Cellular Technology Ltd, S6ULA2-00-6012). Errors were corrected with the counter’s QC program.

### Statistical analysis

All data were analyzed using GraphPad Prism version 9.10. The data were analyzed with a *t* test (∗*p* < 0.05, ∗∗*p* < 0.01, ∗∗∗*p* < 0.001, and ∗∗∗∗*p* < 0.0001). The statistical data are presented as the mean with standard deviation (SD) or median with minimum and maximum values.

## Data and code availability

The datasets generated and analyzed during the current study are available from the corresponding author upon reasonable request. Detailed protocols and supplementary materials supporting the findings of this study will be provided to ensure transparency and reproducibility. Certain data may be subject to restrictions due to ethical considerations or third-party agreements.

## Acknowledgments

The authors thank the Cyagen Biosciences Company for assistance in generating the *Rag2*^*+/pm*^ mice. They also thank Amelia Scheck, Cynthia Klein, and Mark Krampf of Stanford University for their help with laboratory management. They thank Katie Ho and Ethan Haslett for their invaluable assistance and technical expertise throughout this project. Moreover, the authors thank the Stanford Stem Cell Institute FACS Core for flow cytometry access. C.D. was funded by a 10.13039/100015521Stanford Maternal and Child Health Research Institute (MCHRI) postdoctoral training fellowship. This project was funded by a young investigator award to A.C. from the American Society of Transplantation and Cell Therapy (10.13039/100015394ASTCT) and a Cyagen Custom Animal Award to A.C. and 10.13039/100000002National Institutes of Health (NIH) grants R35GM118062, 5R01HL156647, 2RM1HG009490, and 10.13039/100000011HHMI awarded to D.R.L. In addition, flow cytometry instruments were funded by an 10.13039/100000002NIH S10 Shared Instrumentation Grant (1S10RR02933801).

## Author contributions

C.D., J.A.Q., C.T.C., H.N., D.R.L., and A.C. designed the study; C.D., J.A.Q., H.W., C.T.C., L.S., M.D., J.R.D., R.J.M., K.H., M.C., and R.C.W. conducted experiments and analyzed data; C.D., J.A.Q., H.W., R.J.M., and A.C. wrote the manuscript with the input of all authors; A.C. supervised this study.

## Declaration of interests

A.C. and D.R.L. both are inventors on various patents involving antibody conditioning, base editing, and delivery methods. A.C. discloses financial interests in the following entities working in the rare genetic disease space: Beam Therapeutics, Dianthus Therapeutics, Editas Medicines, Fulcrum Therapeutics, GV, Inograft Biotherapeutics, Jasper Therapeutics, Kyowa Kirin, Land Medicine, Prime Medicine, Rocket Pharmaceuticals, STRM.Bio, Spotlight Therapeutics, and Teiko Bio. D.R.L. is a consultant to and equity holder of Prime Medicine, Beam Therapeutics, Pairwise Plants, and nChroma Bio, companies that use gene editing or genome engineering.

## Supplemental Information

Document S1. Figures S1–S5 and Tables S1–S3

Document S2. Article plus supplemental information

## References

[1] Aranda C.S., Gouveia-Pereira M.P., da Silva C.J.M., Rizzo M.C.F.V., Ishizuka E., de Oliveira E.B., Condino-Neto A. (2024). Severe combined immunodeficiency diagnosis and genetic defects. Immunol. Rev..

[2] Hacein-Bey-Abina S., Pai S.Y., Gaspar H.B., Armant M., Berry C.C., Blanche S., Bleesing J., Blondeau J., de Boer H., Buckland K.F. (2014). A modified γ-retrovirus vector for X-linked severe combined immunodeficiency. N. Engl. J. Med..

[3] Schuetz C., Neven B., Dvorak C.C., Leroy S., Ege M.J., Pannicke U., Schwarz K., Schulz A.S., Hoenig M., Sparber-Sauer M. (2014). SCID patients with ARTEMIS vs RAG deficiencies following HCT: increased risk of late toxicity in ARTEMIS-deficient SCID. Blood.

[4] Pai S.Y. (2021). Built to last: gene therapy for ADA SCID. Blood.

[5] Pai S.Y., Logan B.R., Griffith L.M., Buckley R.H., Parrott R.E., Dvorak C.C., Kapoor N., Hanson I.C., Filipovich A.H., Jyonouchi S. (2014). Transplantation outcomes for severe combined immunodeficiency, 2000-2009. N. Engl. J. Med..

[6] Booth C., Kohn D.B., Armant M., Prockop S., Buckland K., Chandra S., Chandrakasan S., Chetty K., Dansereau C., de Oliveira S. (2022). Therapy with Low Dose Conditioning for X-Linked SCID Results in Complete Immune Reconstitution and No Evidence of Clonal Expansion. Blood.

[7] Alsaati N., Grier A., Ochfeld E., McClory S., Heimall J. (2024). Hematopoietic stem cell transplantation for primary immunodeficiency. Allergy Asthma Proc..

[8] Mangum D.S., Caywood E. (2022). A clinician's guide to HLA matching in allogeneic hematopoietic stem cell transplant. Hum. Immunol..

[9] Klein O.R., Bapty S., Lederman H.M., Younger M.E.M., Zambidis E.T., Jones R.J., Cooke K.R., Symons H.J. (2021). Reduced Intensity Bone Marrow Transplantation with Post-Transplant Cyclophosphamide for Pediatric Inherited Immune Deficiencies and Bone Marrow Failure Syndromes. J. Clin. Immunol..

[10] Neven B., Diana J.S., Castelle M., Magnani A., Rosain J., Touzot F., Moreira B., Fremond M.L., Briand C., Bendavid M. (2019). Haploidentical Hematopoietic Stem Cell Transplantation with Post-Transplant Cyclophosphamide for Primary Immunodeficiencies and Inherited Disorders in Children. Biol. Blood Marrow Transpl..

[11] Bordignon C., Mavilio F., Ferrari G., Servida P., Ugazio A.G., Notarangelo L.D., Gilboa E., Rossini S., O'Reilly R.J., Smith C.A. (1993). Transfer of the ADA gene into bone marrow cells and peripheral blood lymphocytes for the treatment of patients affected by ADA-deficient SCID. Hum. Gene Ther..

[12] Kohn D.B., Shaw K.L., Garabedian E., Carbonaro-Sarracino D.A., Moore T.B., De Oliveira S.N., Crooks G.M., Tse J., Shupien S., Terrazas D. (2019). Lentiviral Gene Therapy with Autologous Hematopoietic Stem and Progenitor Cells (HSPCs) for the Treatment of Severe Combined Immune Deficiency Due to Adenosine Deaminase Deficiency (ADA-SCID). Blood.

[13] Cavazzana-Calvo M., Hacein-Bey S., de Saint Basile G., Gross F., Yvon E., Nusbaum P., Selz F., Hue C., Certain S., Casanova J.L. (2000). Gene therapy of human severe combined immunodeficiency (SCID)-X1 disease. Science.

[14] Aiuti A., Slavin S., Aker M., Ficara F., Deola S., Mortellaro A., Morecki S., Andolfi G., Tabucchi A., Carlucci F. (2002). Correction of ADA-SCID by stem cell gene therapy combined with nonmyeloablative conditioning. Science.

[15] David R.M., Doherty A.T. (2017). Viral Vectors: The Road to Reducing Genotoxicity. Toxicol. Sci..

[16] Montini E., Cesana D., Schmidt M., Sanvito F., Bartholomae C.C., Ranzani M., Benedicenti F., Sergi L.S., Ambrosi A., Ponzoni M. (2009). The genotoxic potential of retroviral vectors is strongly modulated by vector design and integration site selection in a mouse model of HSC gene therapy. J. Clin. Invest..

[17] Duncan C.N., Bledsoe J.R., Grzywacz B., Beckman A., Bonner M., Eichler F.S., Kühl J.S., Harris M.H., Slauson S., Colvin R.A. (2024). Hematologic Cancer after Gene Therapy for Cerebral Adrenoleukodystrophy. N. Engl. J. Med..

[18] Schiroli G., Ferrari S., Conway A., Jacob A., Capo V., Albano L., Plati T., Castiello M.C., Sanvito F., Gennery A.R. (2017). Preclinical modeling highlights the therapeutic potential of hematopoietic stem cell gene editing for correction of SCID-X1. Sci. Transl. Med..

[19] De Ravin S.S., Li L., Wu X., Choi U., Allen C., Koontz S., Lee J., Theobald-Whiting N., Chu J., Garofalo M. (2017). CRISPR-Cas9 gene repair of hematopoietic stem cells from patients with X-linked chronic granulomatous disease. Sci. Transl. Med..

[20] Iancu O., Allen D., Knop O., Zehavi Y., Breier D., Arbiv A., Lev A., Lee Y.N., Beider K., Nagler A. (2023). Multiplex HDR for disease and correction modeling of SCID by CRISPR genome editing in human HSPCs. Mol. Ther. Nucleic Acids.

[21] Rai R., Romito M., Rivers E., Turchiano G., Blattner G., Vetharoy W., Ladon D., Andrieux G., Zhang F., Zinicola M. (2020). Targeted gene correction of human hematopoietic stem cells for the treatment of Wiskott - Aldrich Syndrome. Nat. Commun..

[22] Pavel-Dinu M., Wiebking V., Dejene B.T., Srifa W., Mantri S., Nicolas C.E., Lee C., Bao G., Kildebeck E.J., Punjya N. (2019). Author Correction: Gene correction for SCID-X1 in long-term hematopoietic stem cells. Nat. Commun..

[23] Geisinger J.M., Stearns T. (2020). CRISPR/Cas9 treatment causes extended TP53-dependent cell cycle arrest in human cells. Nucleic Acids Res..

[24] Jiang L., Ingelshed K., Shen Y., Boddul S.V., Iyer V.S., Kasza Z., Sedimbi S., Lane D.P., Wermeling F. (2022). CRISPR/Cas9-Induced DNA Damage Enriches for Mutations in a p53-Linked Interactome: Implications for CRISPR-Based Therapies. Cancer Res..

[25] Komor A.C., Kim Y.B., Packer M.S., Zuris J.A., Liu D.R. (2016). Programmable editing of a target base in genomic DNA without double-stranded DNA cleavage. Nature.

[26] Gaudelli N.M., Komor A.C., Rees H.A., Packer M.S., Badran A.H., Bryson D.I., Liu D.R. (2018). Publisher Correction: Programmable base editing of A T to G C in genomic DNA without DNA cleavage. Nature.

[27] Rees H.A., Liu D.R. (2018). Publisher Correction: Base editing: precision chemistry on the genome and transcriptome of living cells. Nat. Rev. Genet..

[28] Chiesa R., Georgiadis C., Syed F., Zhan H., Etuk A., Gkazi S.A., Preece R., Ottaviano G., Braybrook T., Chu J. (2023). Base-Edited CAR7 T Cells for Relapsed T-Cell Acute Lymphoblastic Leukemia. N. Engl. J. Med..

[29] Poli M.C., Aksentijevich I., Bousfiha A.A., Cunningham-Rundles C., Hambleton S., Klein C., Morio T., Picard C., Puel A., Rezaei N. (2025). Human inborn errors of immunity: 2024 update on the classification from the International Union of Immunological Societies Expert Committee. J. Hum. Immun..

[30] Newby G.A., Yen J.S., Woodard K.J., Mayuranathan T., Lazzarotto C.R., Li Y., Sheppard-Tillman H., Porter S.N., Yao Y., Mayberry K. (2021). Base editing of haematopoietic stem cells rescues sickle cell disease in mice. Nature.

[31] Zhang L., Li K., Liu Z., An L., Wei H., Pang S., Cao Z., Huang X., Jin X., Ma X. (2024). Restoring T and B cell generation in X-linked severe combined immunodeficiency mice through hematopoietic stem cells adenine base editing. Mol. Ther..

[32] Gupta A.O., Sharma A., Frangoul H., Dalal J., Kanter J., Alavi A., DiPersio J., Eapen M., Jaroscak J.J., Ayala E. (2024). Initial Results from the BEACON Clinical Study: A Phase 1/2 Study Evaluating the Safety and Efficacy of a Single Dose of Autologous CD34+ Base Edited Hematopoietic Stem Cells (BEAM-101) in Patients with Sickle Cell Disease with Severe Vaso-Occlusive Crises. Blood.

[33] Mangeot P.E., Risson V., Fusil F., Marnef A., Laurent E., Blin J., Mournetas V., Massouridès E., Sohier T.J.M., Corbin A. (2019). Genome editing in primary cells and in vivo using viral-derived Nanoblades loaded with Cas9-sgRNA ribonucleoproteins. Nat. Commun..

[34] Lyu P., Javidi-Parsijani P., Atala A., Lu B. (2019). Delivering Cas9/sgRNA ribonucleoprotein (RNP) by lentiviral capsid-based bionanoparticles for efficient 'hit-and-run' genome editing. Nucleic Acids Res..

[35] Yao X., Lyu P., Yoo K., Yadav M.K., Singh R., Atala A., Lu B. (2021). Engineered extracellular vesicles as versatile ribonucleoprotein delivery vehicles for efficient and safe CRISPR genome editing. J. Extracell. Vesicles.

[36] Hamilton J.R., Tsuchida C.A., Nguyen D.N., Shy B.R., McGarrigle E.R., Sandoval Espinoza C.R., Carr D., Blaeschke F., Marson A., Doudna J.A. (2021). Targeted delivery of CRISPR-Cas9 and transgenes enables complex immune cell engineering. Cell Rep..

[37] Campbell L.A., Coke L.M., Richie C.T., Fortuno L.V., Park A.Y., Harvey B.K. (2019). Gesicle-Mediated Delivery of CRISPR/Cas9 Ribonucleoprotein Complex for Inactivating the HIV Provirus. Mol. Ther..

[38] Geilenkeuser J., Armbrust N., Steinmaßl E., Du S.W., Schmidt S., Binder E.M.H., Li Y., Warsing N.W., Wendel S.V., von der Linde F. (2025). Engineered nucleocytosolic vehicles for loading of programmable editors. Cell.

[39] Han J., Bai H., Li F., Zhang Y., Zhou Q., Li W. (2025). Engineering a streamlined virus-like particle for programmable tissue-specific gene delivery. Nat. Commun..

[40] Botchkarev V.V., Harrington S., Stoppato M., Justen A., Kimber C., Kapuria A., Gibson K.M., Chu C.S., Xu Y., Haugh K. (2025). In vivo gene editing of human hematopoietic stem and progenitor cells using envelope-engineered virus-like particles. Nat. Biotechnol..

[41] Nyberg W.A., Bernard P.L., Ngo W., Wang C.H., Ark J., Rothrock A., Borgo G.M., Kimmerly G.R., Jung J.H., Allain V. (2026). In vivo site-specific engineering to reprogram T cells. Nature.

[42] Villiger L., Rothgangl T., Witzigmann D., Oka R., Lin P.J.C., Qi W., Janjuha S., Berk C., Ringnalda F., Beattie M.B. (2021). In vivo cytidine base editing of hepatocytes without detectable off-target mutations in RNA and DNA. Nat. Biomed. Eng..

[43] Lyu P., Lu Z., Cho S.I., Yadav M., Yoo K.W., Atala A., Kim J.S., Lu B. (2021). Adenine Base Editor Ribonucleoproteins Delivered by Lentivirus-Like Particles Show High On-Target Base Editing and Undetectable RNA Off-Target Activities. CRISPR J..

[44] Choi J.G., Dang Y., Abraham S., Ma H., Zhang J., Guo H., Cai Y., Mikkelsen J.G., Wu H., Shankar P., Manjunath N. (2016). Lentivirus pre-packed with Cas9 protein for safer gene editing. Gene Ther..

[45] Banskota S., Raguram A., Suh S., Du S.W., Davis J.R., Choi E.H., Wang X., Nielsen S.C., Newby G.A., Randolph P.B. (2022). Engineered virus-like particles for efficient in vivo delivery of therapeutic proteins. Cell.

[46] Czechowicz A., Kraft D., Weissman I.L., Bhattacharya D. (2007). Efficient transplantation via antibody-based clearance of hematopoietic stem cell niches. Science.

[47] Bhattacharya D., Czechowicz A., Ooi A.G.L., Rossi D.J., Bryder D., Weissman I.L. (2009). Niche recycling through division-independent egress of hematopoietic stem cells. J. Exp. Med..

[48] Czechowicz A., Bhardwaj R., Park C.Y., Weissman I.L. (2010). Targeted Clearance of Human Hematopoietic Stem Cell Niches Via Inhibition of SCF Signaling Using Monoclonal Antibody SR-1. Blood.

[49] Czechowicz A., Palchaudhuri R., Scheck A., Hu Y., Hoggatt J., Saez B., Pang W.W., Mansour M.K., Tate T.A., Chan Y.Y. (2019). Selective hematopoietic stem cell ablation using CD117-antibody-drug-conjugates enables safe and effective transplantation with immunity preservation. Nat. Commun..

[50] Kwon H.S., Logan A.C., Chhabra A., Pang W.W., Czechowicz A., Tate K., Le A., Poyser J., Hollis R., Kelly B.V. (2019). Anti-human CD117 antibody-mediated bone marrow niche clearance in nonhuman primates and humanized NSG mice. Blood.

[51] Pang W.W., Czechowicz A., Logan A.C., Bhardwaj R., Poyser J., Park C.Y., Weissman I.L., Shizuru J.A. (2019). Anti-CD117 antibody depletes normal and myelodysplastic syndrome human hematopoietic stem cells in xenografted mice. Blood.

[52] Agarwal R., Dvorak C.C., Prohaska S., Long-Boyle J., Kwon H.S., Brown J.W.M., Weinberg K.I., Le A., Guttman-Klein A., Logan A.C. (2019). Toxicity-Free Hematopoietic Stem Cell Engraftment Achieved with Anti-CD117 Monoclonal Antibody Conditioning. Biol. Blood Marrow Transplant..

[53] Agarwal R., Dvorak C.C., Prockop S., Kwon H.S., Long-Boyle J.R., Le A., Brown J.W., Merkel E., Truong K., Velasco B. (2021). JSP191 As a Single-Agent Conditioning Regimen Results in Successful Engraftment, Donor Myeloid Chimerism, and Production of Donor Derived Naïve Lymphocytes in Patients with Severe Combined Immunodeficiency (SCID). Blood.

[54] Clement K., Rees H., Canver M.C., Gehrke J.M., Farouni R., Hsu J.Y., Cole M.A., Liu D.R., Joung J.K., Bauer D.E., Pinello L. (2019). CRISPResso2 provides accurate and rapid genome editing sequence analysis. Nat. Biotechnol..

[55] Ramón-Maiques S., Kuo A.J., Carney D., Matthews A.G.W., Oettinger M.A., Gozani O., Yang W. (2007). The plant homeodomain finger of RAG2 recognizes histone H3 methylated at both lysine-4 and arginine-2. Proc. Natl. Acad. Sci. USA.

[56] Deriano L., Chaumeil J., Coussens M., Multani A., Chou Y., Alekseyenko A.V., Chang S., Skok J.A., Roth D.B. (2011). The RAG2 C terminus suppresses genomic instability and lymphomagenesis. Nature.

[57] Akamatsu Y., Monroe R., Dudley D.D., Elkin S.K., Gartner F., Talukder S.R., Takahama Y., Alt F.W., Bassing C.H., Oettinger M.A. (2003). Deletion of the RAG2 C terminus leads to impaired lymphoid development in mice. Proc. Natl. Acad. Sci. USA.

[58] Girard-Gagnepain A., Amirache F., Costa C., Lévy C., Frecha C., Fusil F., Nègre D., Lavillette D., Cosset F.L., Verhoeyen E. (2014). Baboon envelope pseudotyped LVs outperform VSV-G-LVs for gene transfer into early-cytokine-stimulated and resting HSCs. Blood.

[59] Bernadin O., Amirache F., Girard-Gagnepain A., Moirangthem R.D., Lévy C., Ma K., Costa C., Nègre D., Reimann C., Fenard D. (2019). Baboon envelope LVs efficiently transduced human adult, fetal, and progenitor T cells and corrected SCID-X1 T-cell deficiency. Blood Adv..

[60] Dib C., Queenan J.A., Swartzrock L., Willner H., Denis M., Ahmed N., Moulana Zada F., Borges B., Charlesworth C.T., Lum T. (2025). GFP-on mouse model for interrogation of in vivo gene editing. Nat. Commun..

[61] Lu Z., Yao X., Lyu P., Yadav M., Yoo K., Atala A., Lu B. (2021). Lentiviral Capsid-Mediated. CRISPR J..

[62] An M., Raguram A., Du S.W., Banskota S., Davis J.R., Newby G.A., Chen P.Z., Palczewski K., Liu D.R. (2024). Engineered virus-like particles for transient delivery of prime editor ribonucleoprotein complexes in vivo. Nat. Biotechnol..

[63] Chan Y.Y., Ho P.Y., Swartzrock L., Rayburn M., Nofal R., Thongthip S., Weinberg K.I., Czechowicz A. (2023). Non-genotoxic Restoration of the Hematolymphoid System in Fanconi Anemia. Transpl. Cel. Ther..

[64] Mercier F.E., Sykes D.B., Scadden D.T. (2016). Single Targeted Exon Mutation Creates a True Congenic Mouse for Competitive Hematopoietic Stem Cell Transplantation: The C57BL/6-CD45.1(STEM) Mouse. Stem Cel Rep..

[65] Hou H., Zhou Y., Yu J., Mao L., Bosco M.J., Wang J., Lu Y., Mao L., Wu X., Wang F., Sun Z. (2018). Establishment of the Reference Intervals of Lymphocyte Function in Healthy Adults Based on IFN-γ Secretion Assay upon Phorbol-12-Myristate-13-Acetate/Ionomycin Stimulation. Front. Immunol..

[66] Aiuti A., Cattaneo F., Galimberti S., Benninghoff U., Cassani B., Callegaro L., Scaramuzza S., Andolfi G., Mirolo M., Brigida I. (2009). Gene therapy for immunodeficiency due to adenosine deaminase deficiency. N. Engl. J. Med..

[67] Dvorak C.C., Haddad E., Heimall J., Dunn E., Buckley R.H., Kohn D.B., Cowan M.J., Pai S.Y., Griffith L.M., Cuvelier G.D.E. (2023). The diagnosis of severe combined immunodeficiency (SCID): The Primary Immune Deficiency Treatment Consortium (PIDTC) 2022 Definitions. J. Allergy Clin. Immunol..

[68] Cabeceiras P.K., Adams N., Wang T.D., Woodilla C., Crandall C., Bsharah B., Joung J.K. (2024). Novel Humanized Particles for Efficient Delivery of CRISPR and Other Gene Editors. In MOLECULAR THERAPY.

[69] Montagna C., Petris G., Casini A., Maule G., Franceschini G.M., Zanella I., Conti L., Arnoldi F., Burrone O.R., Zentilin L. (2018). VSV-G-Enveloped Vesicles for Traceless Delivery of CRISPR-Cas9. Mol. Ther. Nucleic Acids.

[70] Strebinger D., Frangieh C.J., Friedrich M.J., Faure G., Macrae R.K., Zhang F. (2023). Cell type-specific delivery by modular envelope design. Nat. Commun..

[71] Igarashi K.J., Kucinski I., Chan Y.Y., Tan T.K., Khoo H.M., Kealy D., Bhadury J., Hsu I., Ho P.Y., Niizuma K. (2023). Physioxia improves the selectivity of hematopoietic stem cell expansion cultures. Blood Adv..

[72] Wilkinson A.C., Ishida R., Kikuchi M., Sudo K., Morita M., Crisostomo R.V., Yamamoto R., Loh K.M., Nakamura Y., Watanabe M. (2019). Author Correction: Long-term ex vivo haematopoietic-stem-cell expansion allows nonconditioned transplantation. Nature.

[73] Bae S., Park J., Kim J.S. (2014). Cas-OFFinder: a fast and versatile algorithm that searches for potential off-target sites of Cas9 RNA-guided endonucleases. Bioinformatics.

[74] Hsu P.D., Scott D.A., Weinstein J.A., Ran F.A., Konermann S., Agarwala V., Li Y., Fine E.J., Wu X., Shalem O. (2013). DNA targeting specificity of RNA-guided Cas9 nucleases. Nat. Biotechnol..

