# Supplementary material for: Restoration of the immune system with base editing and non-genotoxic conditioning in a Rag2 point-mutant mouse model
Source: Mol Ther. 2026 Apr 12;34(7):4070–83. doi: 10.1016/j.ymthe.2026.04.010 (PMC13330013; doi:10.1016/j.ymthe.2026.04.010)
Supplement: Supplementary file 1 [file mmc1.pdf]

YMTHE, Volume *34*

Supplemental Information

Restoration of the immune system with base editing and non-genotoxic conditioning in a *Rag2* point-mutant mouse model

Carla Dib, Jack A. Queenan, Hana Willner, Leah Swartzrock, Carsten T. Charlesworth, Morgane Denis, Jessie R. Davis, Rina J. Mepani, Katie Ho, Madalena Castro, Ross C. Wilson, Hiromitsu Nakauchi, David R. Liu, and Agnieszka Czechowicz

Supplemental Text

The following nucleotide sequences comprise eVLP components optimized for v3b Rag2pm ABE-NG eVLPs and not previously reported for use in eVLPs.

com-sgRNA Rag2^{pm}: Spacer-Scaffold-com-Terminator

GAGGGCTAGCCTAAGAGATCCGTTTAAGAGCTATGCTGGAAACAGCATAGCAAGTTTAAATAAGGCTAGTCCGTTATCAAC
TTGGCTGAATGCCTGCGAGCATCCCACCCAAGTGGCACCGAGTCGGTGCTTTTTTT

P4-ABE8e-NG: P4-Linker-NES-Engineered Protease cleavage site-Linker-FLAG-NLS-ABE8eNG-Linker-NLS

ATGTCTCCCGAAGATAAAATAGCTCAACTCAAACAGAAAATTCAGGCTTTGAAACAAGAAAACCAGCAGTTGGAGGAAGA
AAATGCGGCCCTGGAGTATGGCAGTGGCGGTAGTTCTGGCGGCTCACTTCAACTGCCTCCACTTGAAAGACTGACACTGG
GATCATTACAATTACCTCCTTTAGAACGATTAACACTCGGTTCACTACAGCTTCCGCCTCTTGAGAGATTGACATTAAGTGGT
GGATCTACGTCCACGCTGCTAATGGAGAACTCGTCTGGAGGTTCTGATTACAAAGACGATGACGATAAGAAACGGACAGC
CGACGGAAGCGAGTTCGAGTCACCAAAGAAGAAGCGGAAAGTCTCTGAGGTGGAGTTTTCCCACGAGTACTGGATGAGA
CATGCCCTGACCCTGGCCAAGAGGGCACGGGATGAGAGGGAGGTGCCTGTGGGAGCCGTGCTGGTGCTGAACAATAGA
GTGATCGGCGAGGGCTGGAACAGAGCCATCGGCCTGCACGACCCAACAGCCCATGCCGAAATTATGGCCCTGAGACAG
GGCGGCCTGGTCATGCAGAACTACAGACTGATTGACGCCACCCTGTACGTGACATTCGAGCCTTGCGTGATGTGCGCCG
GCGCCATGATCCACTCTAGGATCGGCCGCGTGGTGTTTGGCGTGAGGAACTCAAAAAGAGGCGCCGCAGGCTCCCTGA
TGAACGTGCTGAACTACCCCGGCATGAATCACCGCGTCGAAATTACCGAGGGAATCCTGGCAGATGAATGTGCCGCCCT
GCTGTGCGATTTCTATCGGATGCCTAGACAGGTGTTCAATGCTCAGAAGAAGGCCCAGAGCTCCATCAACTCCGGAGGAT
CTAGCGGAGGCTCCTCTGGCTCTGAGACACCTGGCACAAGCGAGAGCGCAACACCTGAAAGCAGCGGGGGCAGCAGC
GGGGGGTCAGACAAGAAGTACAGCATCGGCCTGGCCATCGGCACCAACTCTGTGGGCTGGGCCGTGATCACCGACGAG
TACAAGGTGCCCAGCAAGAAATTCAAGGTGCTGGGCAACACCGACCGGCACAGCATCAAGAAGAACCTGATCGGAGCC
CTGCTGTTCGACAGCGGCGAAACAGCCGAGGCCACCCGGCTGAAGAGAACCGCCAGAAGAAGATACACCAGACGGAA
GAACCGGATCTGCTATCTGCAAGAGATCTTCAGCAACGAGATGGCCAAGGTGGACGACAGCTTCTTCCACAGACTGGAA
GAGTCCTTCCTGGTGGAAGAGGATAAGAAGCACGAGCGGCACCCCATCTTCGGCAACATCGTGGACGAGGTGGCCTAC
CACGAGAAGTACCCCACCATCTACCACCTGAGAAAGAAACTGGTGGACAGCACCGACAAGGCCGACCTGCGGCTGATC
TATCTGGCCCTGGCCCACATGATCAAGTTCCGGGGCCACTTCCTGATCGAGGGCGACCTGAACCCCGACAACAGCGAC
GTGGACAAGCTGTTCATCCAGCTGGTGCAGACCTACAACCAGCTGTTCGAGGAAAACCCCATCAACGCCAGCGGCGTG
GACGCCAAGGCCATCCTGTCTGCCAGACTGAGCAAGAGCAGACGGCTGGAAAATCTGATCGCCCAGCTGCCCGGCGA
GAAGAAGAATGGCCTGTTCGGAAACCTGATTGCCCTGAGCCTGGGCCTGACCCCCAACTTCAAGAGCAACTTCGACCT
GGCCGAGGATGCCAAACTGCAGCTGAGCAAGGACACCTACGACGACGACCTGGACAACCTGCTGGCCCAGATCGGCG
ACCAGTACGCCGACCTGTTTCTGGCCGCCAAGAACCTGTCCGACGCCATCCTGCTGAGCGACATCCTGAGAGTGAACA
CCGAGATCACCAAGGCCCCCCTGAGCGCCTCTATGATCAAGAGATACGACGAGCACCACCAGGACCTGACCCTGCTGA
AAGCTCTCGTGCGGCAGCAGCTGCCTGAGAAGTACAAAGAGATTTTCTTCGACCAGAGCAAGAACGGCTACGCCGGCT
ACATTGACGGCGGAGCCAGCCAGGAAGAGTTCTACAAGTTCATCAAGCCCATCCTGGAAAAGATGGACGGCACCGAGG
AACTGCTCGTGAAGCTGAACAGAGAGGACCTGCTGCGGAAGCAGCGGACCTTCGACAACGGCAGCATCCCCCACCAGA
TCCACCTGGGAGAGCTGCACGCCATTCTGCGGCGGCAGGAAGATTTTTACCCATTCCTGAAGGACAACCGGGAAAAGAT
CGAGAAGATCCTGACCTTCCGCATCCCCTACTACGTGGGCCCTCTGGCCAGGGGAAACAGCAGATTCGCCTGGATGACC
AGAAAGAGCGAGGAAACCATCACCCCCTGGAACTTCGAGGAAGTGGTGGACAAGGGCGCTTCCGCCCAGAGCTTCATC
GAGCGGATGACCAACTTCGATAAGAACCTGCCCAACGAGAAGGTGCTGCCCAAGCACAGCCTGCTGTACGAGTACTTCA
CCGTGTATAACGAGCTGACCAAAGTGAAATACGTGACCGAGGGAATGAGAAAGCCCGCCTTCCTGAGCGGCGAGCAGAA
AAAGGCCATCGTGGACCTGCTGTTCAAGACCAACCGGAAAGTGACCGTGAAGCAGCTGAAAGAGGACTACTTCAAGAAA
ATCGAGTGCTTCGACTCCGTGGAAATCTCCGGCGTGGAAGATCGGTTCAACGCCTCCCTGGGCACATACCACGATCTGC
TGAAAATTATCAAGGACAAGGACTTCCTGGACAATGAGGAAAACGAGGACATTCTGGAAGATATCGTGCTGACCCTGACA
CTGTTTGAGGACAGAGAGATGATCGAGGAACGGCTGAAAACCTATGCCCACCTGTTCGACGACAAAGTGATGAAGCAGC
TGAAGCGGCGGAGATACACCGGCTGGGGCAGGCTGAGCCGGAAGCTGATCAACGGCATCCGGGACAAGCAGTCCGGC
AAGACAATCCTGGATTTCCTGAAGTCCGACGGCTTCGCCAACAGAAACTTCATGCAGCTGATCCACGACGACAGCCTGA
CCTTTAAAGAGGACATCCAGAAAGCCCAGGTGTCCGGCCAGGGCGATAGCCTGCACGAGCACATTGCCAATCTGGCCG
GCAGCCCCGCCATTAAGAAGGGCATCCTGCAGACAGTGAAGGTGGTGGACGAGCTCGTGAAAGTGATGGGCCGGCAC
AAGCCCGAGAACATCGTGATCGAAATGGCCAGAGAGAACCAGACCACCCAGAAGGGACAGAAGAACAGCCGCGAGAG
AATGAAGCGGATCGAAGAGGGCATCAAAGAGCTGGGCAGCCAGATCCTGAAAGAACACCCCGTGGAAAACACCCAGCT

GCAGAACGAGAAGCTGTACCTGTACTACCTGCAGAATGGGCGGGATATGTACGTGGACCAGGAACTGGACATCAACCGG
CTGTCCGACTACGATGTGGACCATATCGTGCCTCAGAGCTTTCTGAAGGACGACTCCATCGACAACAAGGTGCTGAC
CAGAAGCGACAAGAACCGGGGCAAGAGCGACAACGTGCCCTCCGAAGAGGTCGTGAAGAAGATGAAGAACTACTG
GCGGCAGCTGCTGAACGCCAAGCTGATTACCCAGAGAAAGTTCGACAATCTGACCAAGGCCGAGAGAGGCGGCCT
GAGCGAACTGGATAAGGCCGGCTTCATCAAGAGACAGCTGGTGGAAACCCGGCAGATCACAAAGCACGTGGCACAG
ATCCTGGACTCCCGGATGAACACTAAGTACGACGAGAATGACAAGCTGATCCGGGAAGTGAAAGTGATCACCCTGAAG
TCCAAGCTGGTGTCCGATTTCCGGAAGGATTTCCAGTTTTACAAAGTGCGCGAGATCAACAACTACCACCACGCCCAC
GACGCCTACCTGAACGCCGTCGTGGGAACCGCCCTGATCAAAAAGTACCCTAAGCTGGAAAGCGAGTTCGTGTACGG
CGACTACAAGGTGTACGACGTGCGGAAGATGATCGCCAAGAGCGAGCAGGAAATCGGCAAGGCTACCGCCAAGTACT
TCTTCTACAGCAACATCATGAACTTTTTCAAGACCGAGATTACCCTGGCCAACGGCGAGATCCGGAAGCGGCCTCTGAT
CGAGACAAACGGCGAAACCGGGGAGATCGTGTGGGATAAGGGCCGGGATTTTGCCACCGTGCGGAAAGTGCTGAGCA
TGCCCCAAGTGAATATCGTGAAAAAGACCGAGGTGCAGACAGGCGGCTTCAGCAAAGAGTCTATCAGGCCCAAGAGGA
ACAGCGATAAGCTGATCGCCAGAAAGAAGGACTGGGACCCTAAGAAGTACGGCGGCTTCGTCAGCCCCACCGTGGCCT
ATTCTGTGCTGGTGGTGGCCAAAGTGGAAAAGGGCAAGTCCAAGAAACTGAAGAGTGTGAAAGAGCTGCTGGGGATCA
CCATCATGGAAAGAAGCAGCTTCGAGAAGAATCCCATCGACTTTCTGGAAGCCAAGGGCTACAAAGAAGTGAAAAAGGA
CCTGATCATCAAGCTGCCTAAGTACTCCCTGTTCGAGCTGGAAAACGGCCGGAAGAGAATGCTGGCCTCTGCCAGATTC
CTGCAGAAGGGAAACGAACTGGCCCTGCCCTCCAAATATGTGAACTTCCTGTACCTGGCCAGCCACTATGAGAAGCTGA
AGGGCTCCCCCGAGGATAATGAGCAGAAACAGCTGTTTGTGGAACAGCACAAGCACTACCTGGACGAGATCATCGAGC
AGATCAGCGAGTTCTCCAAGAGAGTGATCCTGGCCGACGCTAATCTGGACAAAGTGCTGTCCGCCTACAACAAGCACCG
GGATAAGCCCATCAGAGAGCAGGCCGAGAATATCATCCACCTGTTTACCCTGACCAATCTGGGAGCCCCTAGGGCCTTCA
AGTACTTTGACACCACCATCGACCGGAAGGTGTACAGGAGCACCAAAGAGGTGCTGGACGCCACCCTGATCCACCAGA
GCATCACCGGCCTGTACGAGACACGGATCGACCTGTCTCAGCTGGGAGGTGACTCTGGCGGCTCAAAAAGAACCGCC
GACGGCAGCGAATTCGAGCCCAAGAAGAAGAGGAAAGTCTGA

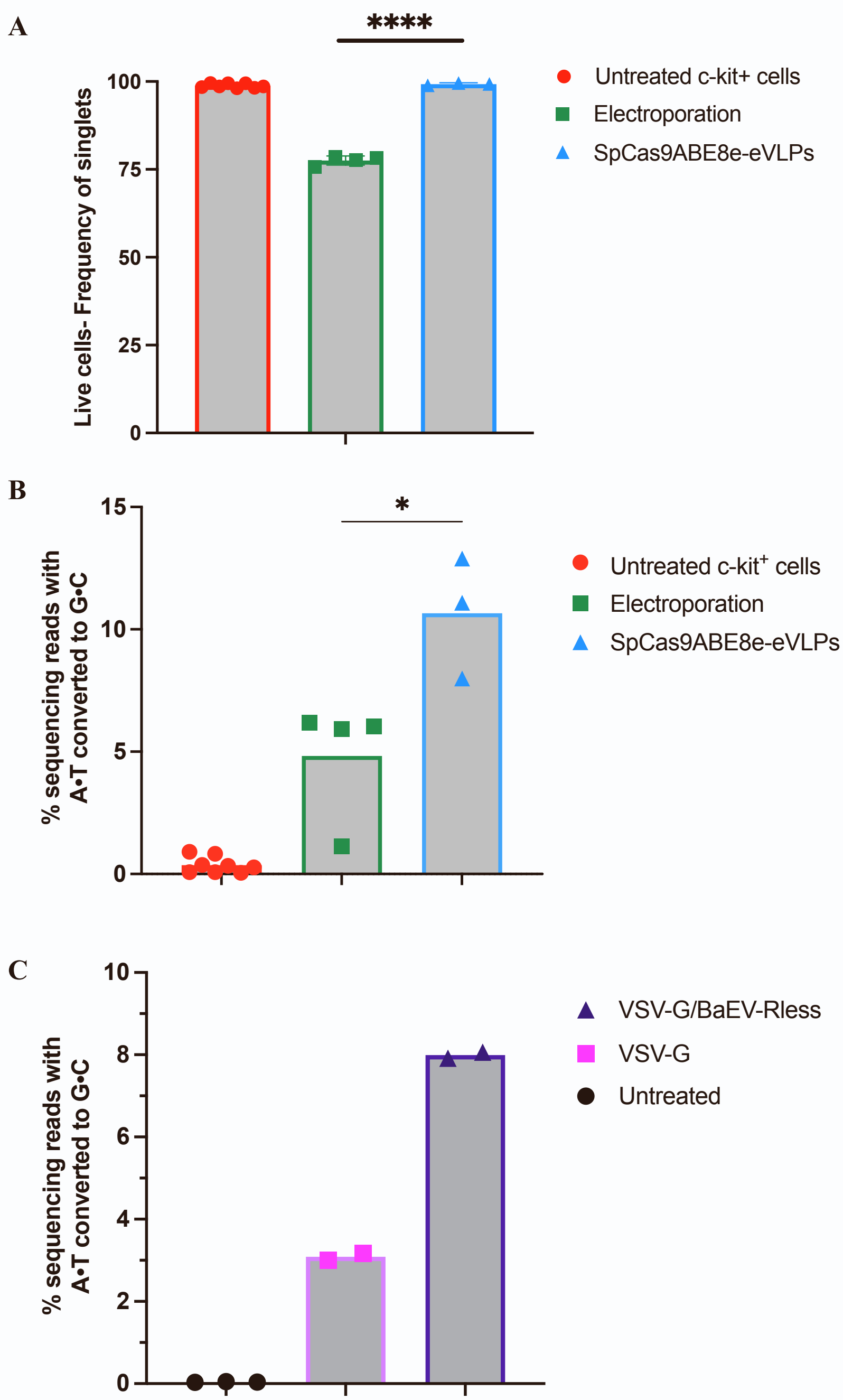

Figure S1: ABE-eVLP transduction of *Rag2*$^{pm/pm}$ c-kit$^+$ HSPCs. (A) Cell viability and **(B)** adenine base editing efficiency 24h following ABE-RNP electroporation or v3b ABE-eVLP delivery. **(C)** Base editing efficiency 24h following v3b ABE-eVLP delivery from eVLPs pseudotyped with VSV-G alone or co-pseudotyped with HSC-tropic BaEV-Rless envelope.

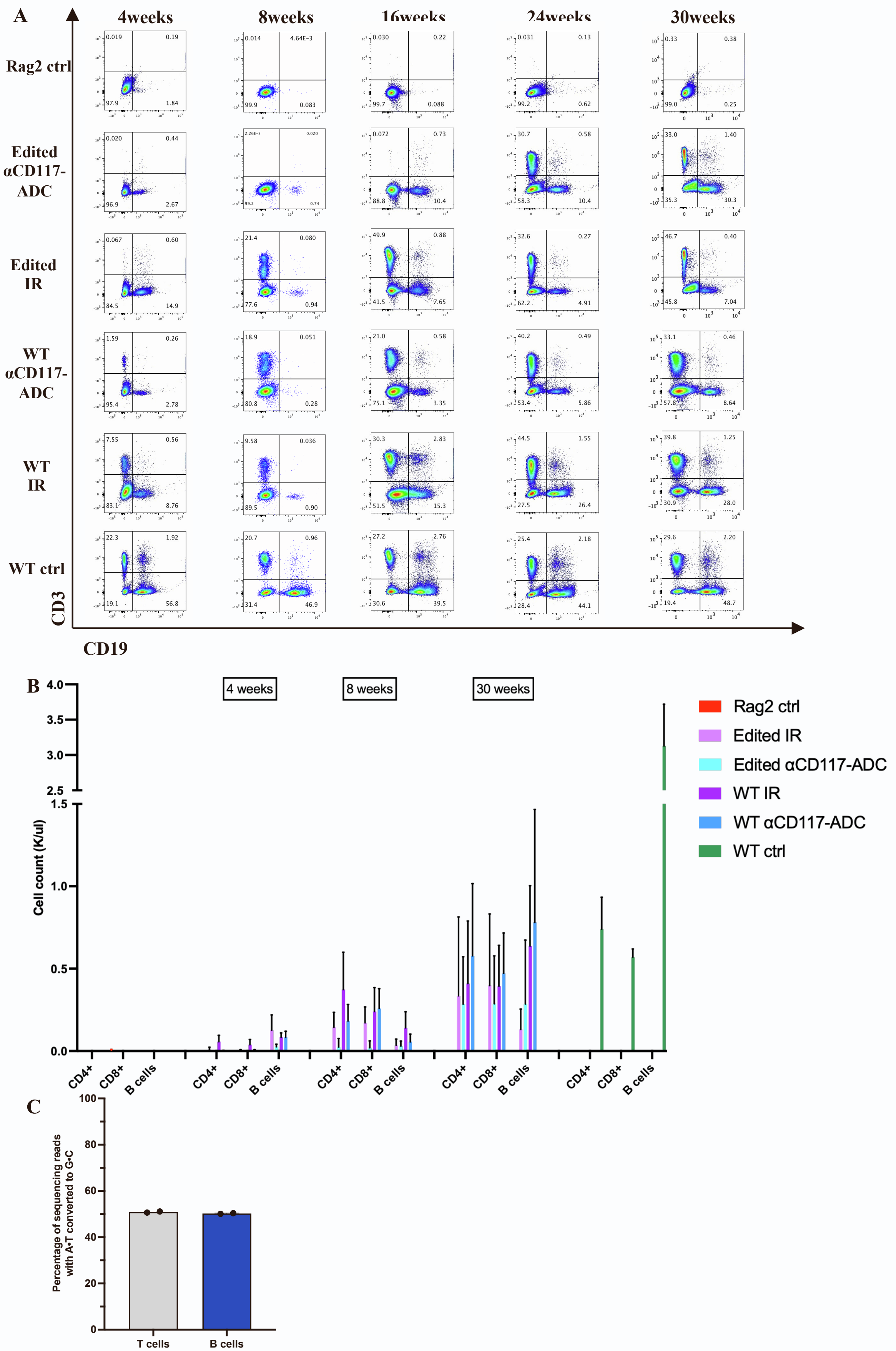

Figure S2: SCID phenotype reversion in transplanted *Rag2$^{pm/pm}$* mice. (A) Representative flow cytometry plots of peripheral blood show long-term production of T- and B-cells in transplanted mice compared to untreated *Rag2$^{pm/pm}$* and WT controls. **(B)** Lymphoid lineage counts of CD4^{+} T-cells, CD8^{+} T-cells and B-cells steadily increased in all transplanted mice and at 30 weeks post-transplant approached levels in untreated WT controls. **(C)** HTS analysis shows 50% of edited alleles in B- and T-cells sorted from mice transplanted with v3b-transduced HSPCs at 16 weeks post-transplantation.

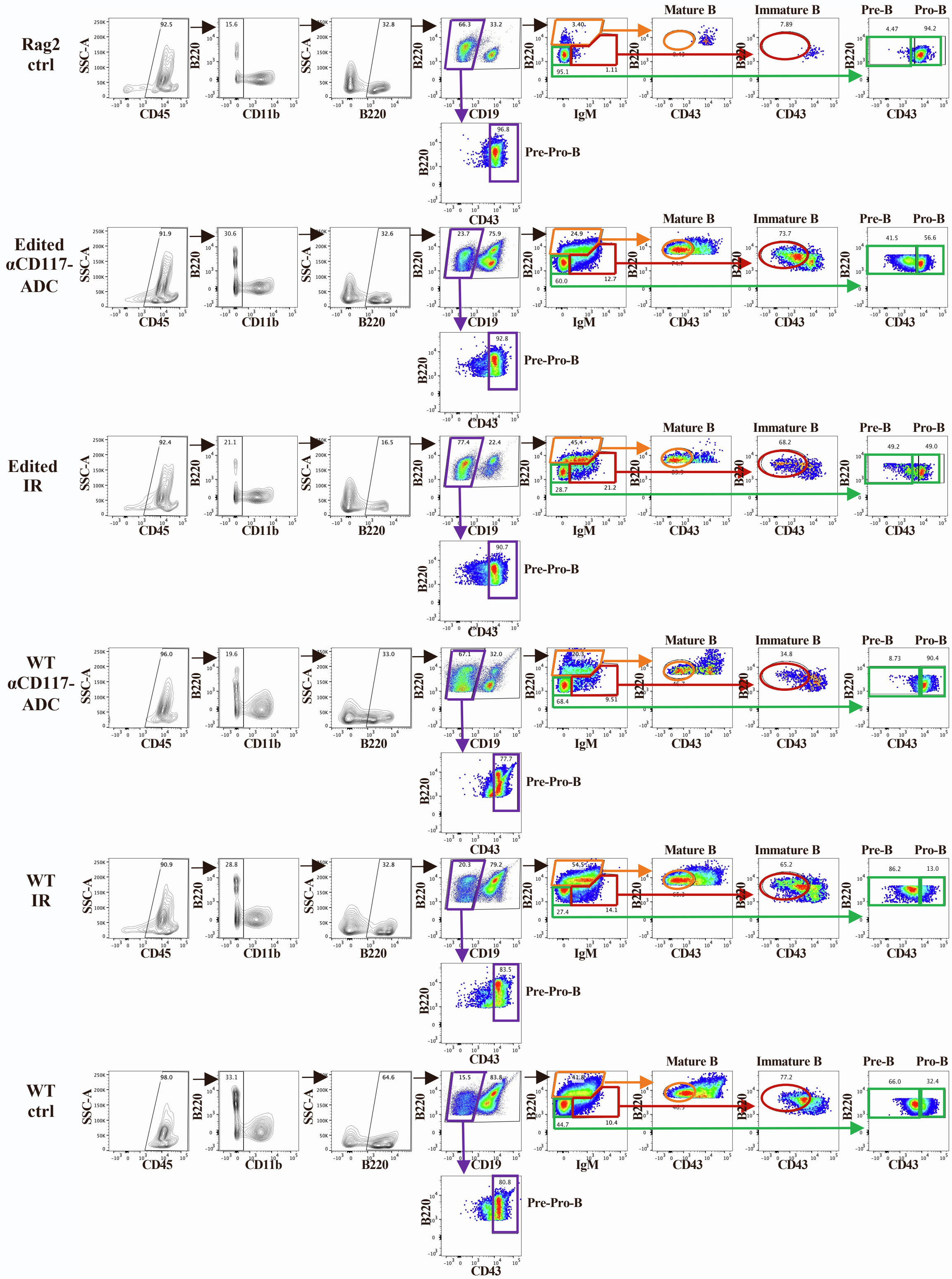

Figure S3: B-cell progenitor differentiation corrected in transplanted *Rag2^pm/pm^* mice. Representative flow cytometry plots show B-progenitors in bone marrow with normal progression across all differentiation stages in transplanted mice compared to untreated *Rag2^pm/pm^* and WT controls.

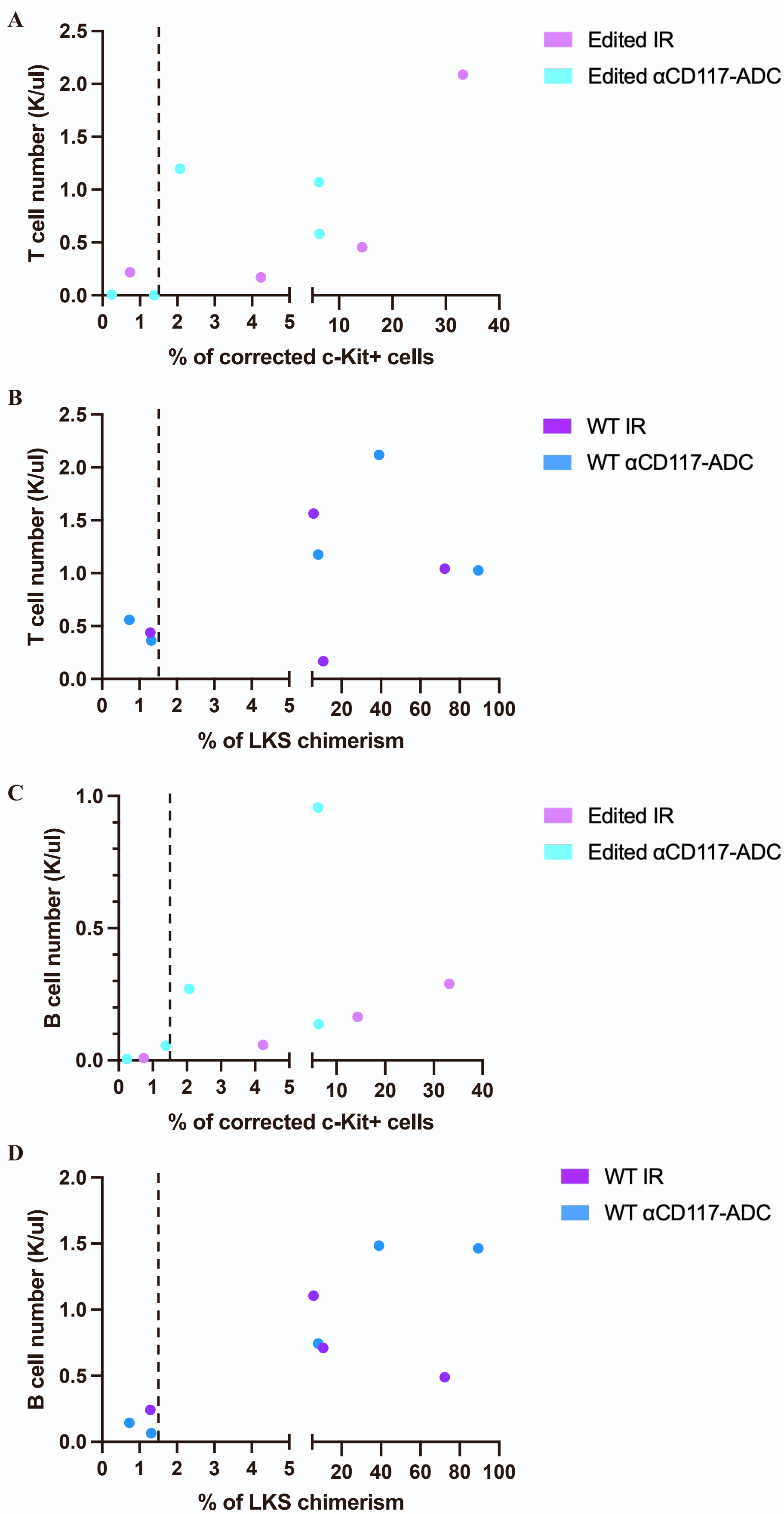

Figure S4: Engraftment of varying frequencies of edited or wild-type (WT) HSPCs in *Rag2*$^{pm/pm}$ mice results in T-cell and B-cell production. T-cell production was observed in mice transplanted with both **(A)** edited and **(B)** WT HSPCs. **(C-D)** Similarly, B-cell production was also observed in these same animals. All mice with > 1.5% edited or WT HSPCs in the bone marrow (dashed line) had robust T- and B-cell production, while those with less had varying results.

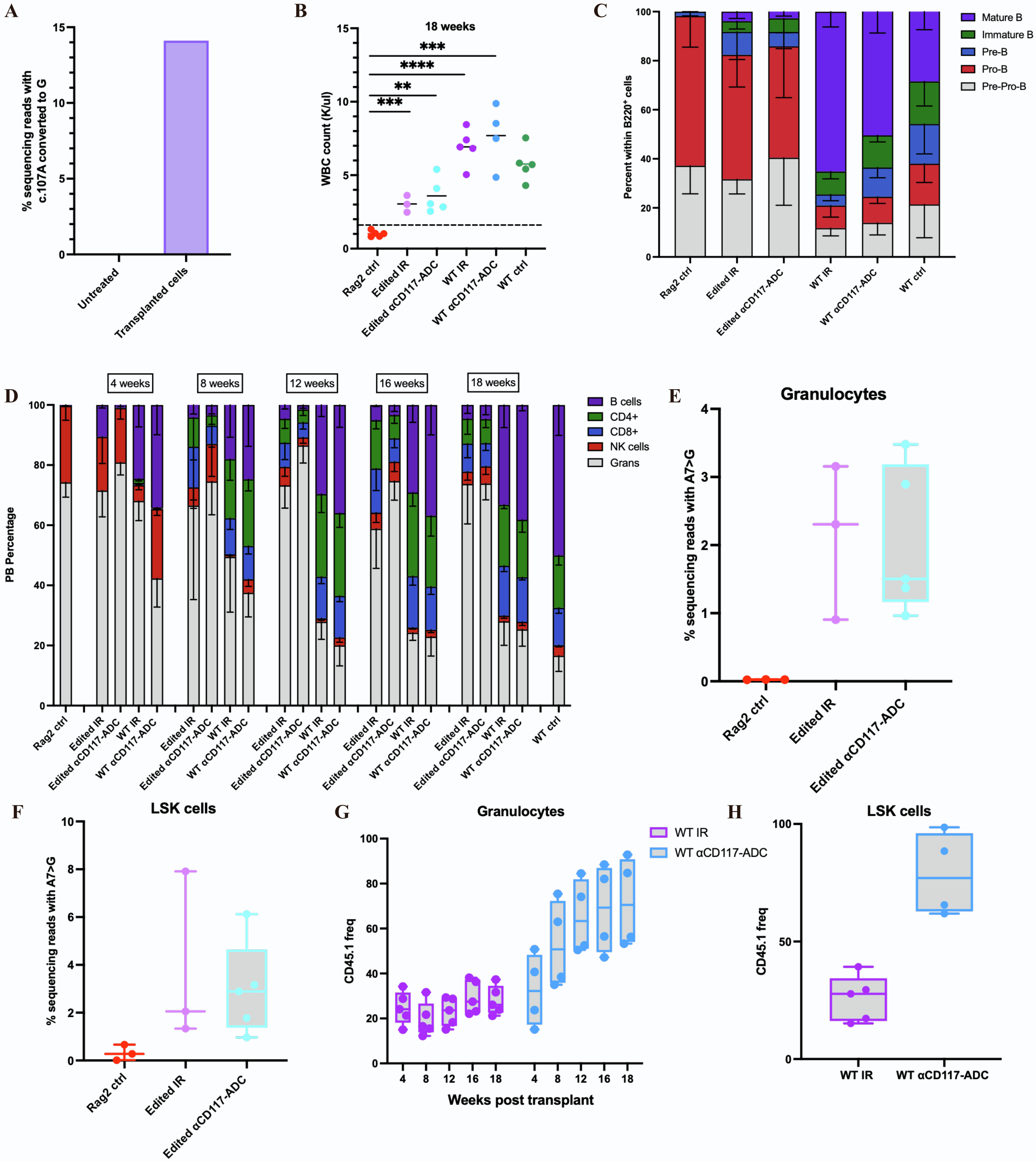

Figure S5: Replicate experiment exhibiting T-cell and B-cell immune reconstitution of *Rag2$^{pm/pm}$* mice transplanted with ABE-eVLP-transduced *Rag2$^{pm/pm}$* HSPCs or wild-type (WT) HSPCs post irradiation (IR) or αCD117-ADC conditioning. (A) ABE-eVLP transduction resulted in ~14% correction of c-kit+ HSPCs from *Rag2$^{pm/pm}$* mice which were subsequently transplanted into conditioned *Rag2$^{pm/pm}$* mice. **(B)** At 18 weeks post-transplantation, the WBC count was higher in all transplanted mice compared to untransplanted controls (dashed line). **(C)** Percentage composition of B-cell progenitors in the bone marrow at 18 weeks post-transplantation. Error bars: SD. **(D)** T- and B-cell production was observed in transplanted mice, beginning at 4 weeks and continuing until 18 weeks post-transplantation, as assessed by flow cytometry. Error bars: SD. Genetic correction in **(E)** granulocytes and **(F)** LSK cells at 18 weeks post-transplantation obtained from *Rag2$^{pm/pm}$* mice transplanted with ABE-eVLP-transduced *Rag2$^{pm/pm}$* HSPCs as assessed by HTS. Donor **(G)** granulocyte and **(H)** LSK chimerism at 18 weeks post-transplantation in *Rag2$^{pm/pm}$* mice transplanted with WT CD45.1 HSPCs as evaluated by flow cytometry. Boxplots show the median, range, and quartiles.

Table S1: Computationally nominated off-target sites.

Amplicon	gRNA	DNA	Genomic Coordinate (mm10)	Locus Details
OT1	AGGGCTAGCCTAAGAGATCC	tGGaCTA-CCTAAGAGATCC	chr6:116237205-116237223	Intronic/Intergenic
OT2	AGGGCTAGCCTAAGAGATCC	AGGGCTAGgCTAAGAG-TCC	chr4:58871525-588715443	Intronic/Intergenic
OT3	AGGGCTAGCCTAAGAGATCC	tGGaCT-GCCTAAGAGATCC	chr11:4723338-4723356	Intronic/Intergenic
OT4	AGGGCTAGCCTAAGAGATCC	gaGGCT-GCCTAAGAGATCC	chr13:103664418-103664436	Intronic/Intergenic
OT5	AGGGCTAGCCTAAGAGATCC	A-GGCTAttCTAAGAGATCC	chr18:68392222-68392240	Intronic/Intergenic
OT6	AGGGCTAGCCTAAGAGATCC	AGaG-TAGCCcAAGAGATCC	chr8:30778108-30778126	Intronic/Intergenic
OT7	AGGGCTAGCCTAAGAGATCC	AGGaaTAGCCaAAGAGATCC	chr13:52521186-52521205	Intronic/Intergenic
OT8	AGGGCTAGCCTAAGAGATCC	AGaGCTA-CCTAAGAGATCg	chr11:95073378-95073396	Intronic/Intergenic
OT9	AGGGCTAGCCTAAGAGATCC	AGGaCaAGC-TAAGAGATCC	chr3:38482754-38482772	Exonic (*Ankrd50*)
OT10	AGGGCTAGCCTAAGAGATCC	caGG-TAaCCTAAGAGATCC	chr15:76996780-76996798	Intronic/Intergenic
OT11	AGGGCTAGCCTAAGAGATCC	AaGGCTAGC-TAAGAGATCt	chr2:35606314-35606332	Intronic/Intergenic
OT12	AGGGCTAGCCTAAGAGATCC	AatcCTA-CCTAAGAGATCC	chr8:43545374-43545392	Intronic/Intergenic
OT13	AGGGCTAGCCTAAGAGATCC	AGGGtTA-CCTgAGAGATCC	chr16:38033372-38033390	Intronic/Intergenic
OT14	AGGGCTAGCCTAAGAGATCC	AGtGtTAaCaTAAGAGATCC	chr9:24492794-24492813	Intronic/Intergenic
OT15	AGGGCTAGCCTAAGAGATCC	AtGGCTA-CCTAAGAGtTCC	chr16:39462242-39462260	Intronic/Intergenic
OT16	AGGGCTAGCCTAAGAGATCC	AGaGCTA-CCTAAGAGcTCC	chr14:57503099-57503117	Intronic/Intergenic
OT17	AGGGCTAGCCTAAGAGATCC	AGGG-TAGCaTgAGAGATCC	chr14:71144536-71144554	Intronic/Intergenic
OT18	AGGGCTAGCCTAAGAGATCC	AtGGCTtGCCaAAGAGATCC	chr6:80097817-80097836	Intronic/Intergenic
OT19	AGGGCTAGCCTAAGAGATCC	AGGGtTgGCC-AAGAGATCC	chr12:15616860-15616878	Intronic/Intergenic
OT20	AGGGCTAGCCTAAGAGATCC	A-GGCTgGCCTcAGAGATCC	chr7:27496082-27496100	Intronic/Intergenic
OT21	AGGGCTAGCCTAAGAGATCC	ctGG-TgGCCTAAGAGATCC	chr15:54961370-54961388	Intronic/Intergenic
OT22	AGGGCTAGCCTAAGAGATCC	AaG-CTgaCCTAAGAGATCC	chr5:106655314-106655332	Intronic/Intergenic
OT23	AGGGCTAGCCTAAGAGATCC	A-GGaTgaCCTAAGAGATCC	chr2:145615241-145615259	Intronic/Intergenic
OT24	AGGGCTAGCCTAAGAGATCC	AGGGCT-GtaTAAGAGATCC	chr9:120762092-120762110	Intronic/Intergenic
OT25	AGGGCTAGCCTAAGAGATCC	AGtcaTtGCCTAAGAGATCC	chr3:3507551-3507570	Intronic/Intergenic
OT26	AGGGCTAGCCTAAGAGATCC	AGGcCaAGCC-AAGAGATCC	chr14:30036928-30036946	Intronic/Intergenic
OT27	AGGGCTAGCCTAAGAGATCC	AGGGtcAGCCaAAGAGATCC	chrX:60791023-60791042	Intronic/Intergenic
OT28	AGGGCTAGCCTAAGAGATCC	AGGGtTtaCtTAAGAGATCC	chr7:103828622-103828641	Intronic/Intergenic
OT29	AGGGCTAGCCTAAGAGATCC	AGGG-TcaCgTAAGAGATCC	chr2:163473680-163473698	Intronic/Intergenic
OT30	AGGGCTAGCCTAAGAGATCC	AaGGCcAGCCTgAGAGATCC	chr5:99631853-99631872	Intronic/Intergenic
OT31	AGGGCTAGCCTAAGAGATCC	AcaGCaAcCCTAAGAGATCC	chr13:14655542-14655561	Intronic/Intergenic
OT32	AGGGCTAGCCTAAGAGATCC	gGGcCTAcCCaAAGAGATCC	chr5:25412707-25412726	Intronic/Intergenic
OT33	AGGGCTAGCCTAAGAGATCC	AGGGCTAtaC-AAGAGATCC	chr1:115664044-115664062	Intronic/Intergenic
OT34	AGGGCTAGCCTAAGAGATCC	AGGGCTAcaCaAAGAGATCC	chr4:151959265-151959284	Intronic/Intergenic
OT35	AGGGCTAGCCTAAGAGATCC	AGGGCTAGggaAAGAGATCC	chr11:68274903-68274922	Intronic/Intergenic
OT36	AGGGCTAGCCTAAGAGATCC	gtGGCTAGCCTAAGAGAaCC	chr5:33031858-33031877	Intronic/Intergenic

Table S2: HTS oligonucleotide primers.

Name	Sequence
mRag2pm HTS F	ACACTCTTTCCCTACACGACGCTCTTCCGATCTNNNNCCCTGCAGATGGTAACAGTGGG
mRag2pm HTS R	TGGAGTTCAGACGTGTGCTCTTCCGATCTTTGATGCTTGTCAGAGTCTATGC
mRag2 OT1 HTS F	ACACTCTTTCCCTACACGACGCTCTTCCGATCTNNNNTACAAAGGCATGGAACGGTTT
mRag2 OT1 HTS R	TGGAGTTCAGACGTGTGCTCTTCCGATCTACCAAACCTGGGCTCTCTGAT
mRag2 OT2 HTS F	ACACTCTTTCCCTACACGACGCTCTTCCGATCTNNNNCAGCACCAGATTATGAAACAGAC
mRag2 OT2 HTS R	TGGAGTTCAGACGTGTGCTCTTCCGATCTCCACTCTAATGAGTGTGACATGACC
mRag2 OT3 HTS F	ACACTCTTTCCCTACACGACGCTCTTCCGATCTNNNNATTGTTTGCCCAGCACGCAG
mRag2 OT3 HTS R	TGGAGTTCAGACGTGTGCTCTTCCGATCTCCATGCTTCTCTTACAGGCCG
mRag2 OT4 HTS F	ACACTCTTTCCCTACACGACGCTCTTCCGATCTNNNNACTGTCCCTCAGCCAAGTCAT
mRag2 OT4 HTS R	TGGAGTTCAGACGTGTGCTCTTCCGATCTCCAGAGGTCTCCAGCAGTTTG
mRag2 OT5 HTS F	ACACTCTTTCCCTACACGACGCTCTTCCGATCTNNNNACTCACTAAAGGTGGGAAAGGACT
mRag2 OT5 HTS R	TGGAGTTCAGACGTGTGCTCTTCCGATCTTCTCCAGTCCCAGAGATGTGA
mRag2 OT6 HTS F	ACACTCTTTCCCTACACGACGCTCTTCCGATCTNNNNATGGGGCTTCTGTGAAATGC
mRag2 OT6 HTS R	TGGAGTTCAGACGTGTGCTCTTCCGATCTTTCATCCCCTGATGCTCCAA
mRag2 OT7 HTS F	ACACTCTTTCCCTACACGACGCTCTTCCGATCTNNNNCTAGTCCCCTGGCCCACGAA
mRag2 OT7 HTS R	TGGAGTTCAGACGTGTGCTCTTCCGATCTCTGCCGATGCCCCACTGTTT
mRag2 OT8 HTS F	ACACTCTTTCCCTACACGACGCTCTTCCGATCTNNNNAGGAGCAGCTCTTCTCCCTC
mRag2 OT8 HTS R	TGGAGTTCAGACGTGTGCTCTTCCGATCTAAAGCCAAAGCCTCCCCCA
mRag2 OT9 HTS F	ACACTCTTTCCCTACACGACGCTCTTCCGATCTNNNNAGAGAGGTCTGCATCGCCAG
mRag2 OT9 HTS R	TGGAGTTCAGACGTGTGCTCTTCCGATCTCAAGCATGGCTACCTTCCCA
mRag2 OT10 HTS F	ACACTCTTTCCCTACACGACGCTCTTCCGATCTNNNNTCCACCTAGGCAAACACCCT
mRag2 OT10 HTS R	TGGAGTTCAGACGTGTGCTCTTCCGATCTGGCGGTGCAGGCTTTTTATTG
mRag2 OT12 HTS F	ACACTCTTTCCCTACACGACGCTCTTCCGATCTNNNNCTAACTGGGGATGCTTCGGC
mRag2 OT12 HTS R	TGGAGTTCAGACGTGTGCTCTTCCGATCTAACTAGATGTGCCAAAGGGAC
mRag2 OT13 HTS F	ACACTCTTTCCCTACACGACGCTCTTCCGATCTNNNNTGAGAAAGCAGGCCAAGCCAT
mRag2 OT13 HTS R	TGGAGTTCAGACGTGTGCTCTTCCGATCTGTTCTCGGACCCAGATTTCTTTTC
mRag2 OT14 HTS F	ACACTCTTTCCCTACACGACGCTCTTCCGATCTNNNNAGCCAAACAGTACTGGCCACC
mRag2 OT14 HTS R	TGGAGTTCAGACGTGTGCTCTTCCGATCTACCTGAGCTCTCTTCCGTGGT
mRag2 OT15 HTS F	ACACTCTTTCCCTACACGACGCTCTTCCGATCTNNNNAGATGCGCAGCATTGACATTAC
mRag2 OT15 HTS R	TGGAGTTCAGACGTGTGCTCTTCCGATCTTCTCATTTCATCCTTTCATCACTGC
mRag2 OT16 HTS F	ACACTCTTTCCCTACACGACGCTCTTCCGATCTNNNNATCTCAGAGGTGCCTGGGCT
mRag2 OT16 HTS R	TGGAGTTCAGACGTGTGCTCTTCCGATCTAGGGCAGAGCAGAGACGAGT
mRag2 OT17 HTS F	ACACTCTTTCCCTACACGACGCTCTTCCGATCTNNNNTGCTTACTGGTTGCCAGGCG
mRag2 OT17 HTS R	TGGAGTTCAGACGTGTGCTCTTCCGATCTAGAGTACACTGCCCCAGACGG
mRag2 OT18 HTS F	ACACTCTTTCCCTACACGACGCTCTTCCGATCTNNNNAACTGGGTGTAGCGAAGCGG
mRag2 OT18 HTS R	TGGAGTTCAGACGTGTGCTCTTCCGATCTCTCCTGCGGCACGCATTCTC
mRag2 OT19 HTS F	ACACTCTTTCCCTACACGACGCTCTTCCGATCTNNNNCATGGCTTCCAAGCTCCCTT
mRag2 OT19 HTS R	TGGAGTTCAGACGTGTGCTCTTCCGATCTAACATAACATTGTCCTTTTGCTACT
mRag2 OT20 HTS F	ACACTCTTTCCCTACACGACGCTCTTCCGATCTNNNNACCGCTGAGCCATTCTCTCC
mRag2 OT20 HTS R	TGGAGTTCAGACGTGTGCTCTTCCGATCTGCCAACCTGCTTTTGGGGTC
mRag2 OT21 HTS F	ACACTCTTTCCCTACACGACGCTCTTCCGATCTNNNNAGCCCTAGGATGACAGTGAGGAT
mRag2 OT21 HTS R	TGGAGTTCAGACGTGTGCTCTTCCGATCTTGGCCAGAGGGCAGGAATTATTTT
mRag2 OT22 HTS F	ACACTCTTTCCCTACACGACGCTCTTCCGATCTNNNNAAAGCGCCATTCTCTGGGC
mRag2 OT22 HTS R	TGGAGTTCAGACGTGTGCTCTTCCGATCTGATCTCTGTGAGTTGGAGGCCA
mRag2 OT23 HTS F	ACACTCTTTCCCTACACGACGCTCTTCCGATCTNNNNAGGGTGGACTCCCTGTGACC
mRag2 OT23 HTS R	TGGAGTTCAGACGTGTGCTCTTCCGATCTAGGGAGCTGGCAAGAGAGGT
mRag2 OT24 HTS F	ACACTCTTTCCCTACACGACGCTCTTCCGATCTNNNNAGTTGGCCTATGAGATGGGCT
mRag2 OT24 HTS R	TGGAGTTCAGACGTGTGCTCTTCCGATCTATTGATATTTTGCTTGCATGAGTGT
mRag2 OT25 HTS F	ACACTCTTTCCCTACACGACGCTCTTCCGATCTNNNNGCCTTCTTCCTTCCCTGCCC
mRag2 OT25 HTS R	TGGAGTTCAGACGTGTGCTCTTCCGATCTTTCCTTTCCCCTCCCCTCGG
mRag2 OT27 HTS F	ACACTCTTTCCCTACACGACGCTCTTCCGATCTNNNNCAGCCATGAGCATAACGCCT
mRag2 OT27 HTS R	TGGAGTTCAGACGTGTGCTCTTCCGATCTGAGGGTCTGAAGAGGGAGAAAAGC
mRag2 OT28 HTS F	ACACTCTTTCCCTACACGACGCTCTTCCGATCTNNNNTGTGTGGGAGGAGTGTACAAGGA
mRag2 OT28 HTS R	TGGAGTTCAGACGTGTGCTCTTCCGATCTGGGCAGCATGTGCTGAGGA
mRag2 OT30 HTS F	ACACTCTTTCCCTACACGACGCTCTTCCGATCTNNNNACCACCTGTCTACTTCTTGGC
mRag2 OT30 HTS R	TGGAGTTCAGACGTGTGCTCTTCCGATCTGTAGCAGTGAAGGTCAGCGG
mRag2 OT32 HTS F	ACACTCTTTCCCTACACGACGCTCTTCCGATCTNNNNAACCCTTGTCTAATACCCCATGC
mRag2 OT32 HTS R	TGGAGTTCAGACGTGTGCTCTTCCGATCTGGCAGTGGTACCCTATGCCTT
mRag2 OT33 HTS F	ACACTCTTTCCCTACACGACGCTCTTCCGATCTNNNNACTCTGCAAGTTGTGACAGGTG
mRag2 OT33 HTS R	TGGAGTTCAGACGTGTGCTCTTCCGATCTAAGAGCTGGACAGTGGTGGC
mRag2 OT34 HTS F	ACACTCTTTCCCTACACGACGCTCTTCCGATCTNNNNAGCCACCATGTGGGTGCTA
mRag2 OT34 HTS R	TGGAGTTCAGACGTGTGCTCTTCCGATCTCCCAGCTGGAGGCAGAGTATTT
mRag2 OT35 HTS F	ACACTCTTTCCCTACACGACGCTCTTCCGATCTNNNNCTGTGAGGGCCTAGCATGGG
mRag2 OT35 HTS R	TGGAGTTCAGACGTGTGCTCTTCCGATCTGAGGGGTGTGGCTCTGAATC
mRag2 OT36 HTS F	ACACTCTTTCCCTACACGACGCTCTTCCGATCTNNNNGTCCCCCTCCTGGGAAACTT
mRag2 OT36 HTS R	TGGAGTTCAGACGTGTGCTCTTCCGATCTAGTCTTGCAGCACCAATCCTT

Table S3: Flow cytometry antibodies.

Antibody	Antibody clone	Identifier
CD3	17A2	Cat# 100246
CD4	RM4-5	Cat# 100516
CD8	53-6.7	Cat# 100722
Gr1	RB6-8C5	Cat# 108424
Nk1.1	PK136	Cat# 108716
CD19	MB19-1	Cat# 11-0191-85
B220	RA3-6B2	Cat# 103255
CD45.1	A20	Cat# 110722
CD45.2	104	Cat# 109841
Ter119	TER-119	Cat# 116220
Lineage	17A2, RB6-8C5, RA3-6B2, Ter-119, M1/70	Cat# 133313
CD117	2B8	Cat# 105808
Sca1	D7	Cat# 108112
CD48	HM48-1	Cat# 103432
CD150	TC15-12F12.2	Cat# 115914
CD45	30-F11	Cat# 103112
IgM	RMM-1	Cat# 406506
CD11b	M1/70	Cat# 101208
CD43	S11	Cat# 143210
